# Supplementary material for: Iridium-Catalyzed Synthesis of Chiral 1,2,3-Triazoles Units and Precise Construction of Stereocontrolled Oligomers
Source: Molecules. 2023 Apr 26;28(9):3726. doi: 10.3390/molecules28093726 (PMC10180159; doi:10.3390/molecules28093726)
Supplement: Supplementary file 1 [file molecules-28-03726-s001.zip › molecules-2353499-supplementary.pdf]

Supplementary Information

Iridium-Catalyzed Synthesis of Chiral 1,2,3-Triazoles Units and Precise Construction of Stereocontrolled Oligomers

Xueyan Zhang,[1] Tian Yu,[1] Shengtao Ding[1,2,*]

[1] *State Key Laboratory of Organic-Inorganic Composites, College of Chemical Engineering, Beijing University of Chemical Technology, Beijing 100029, China.*

[2] *State Key Laboratory of Natural and Biomimetic Drugs, School of Pharmaceutical Sciences, Peking University, Beijing, 100191, China.*

Table of Contents

I. ^{1}H NMR Spectra 2

II. MS spectra 31

I. ^{1}H NMR Spectra

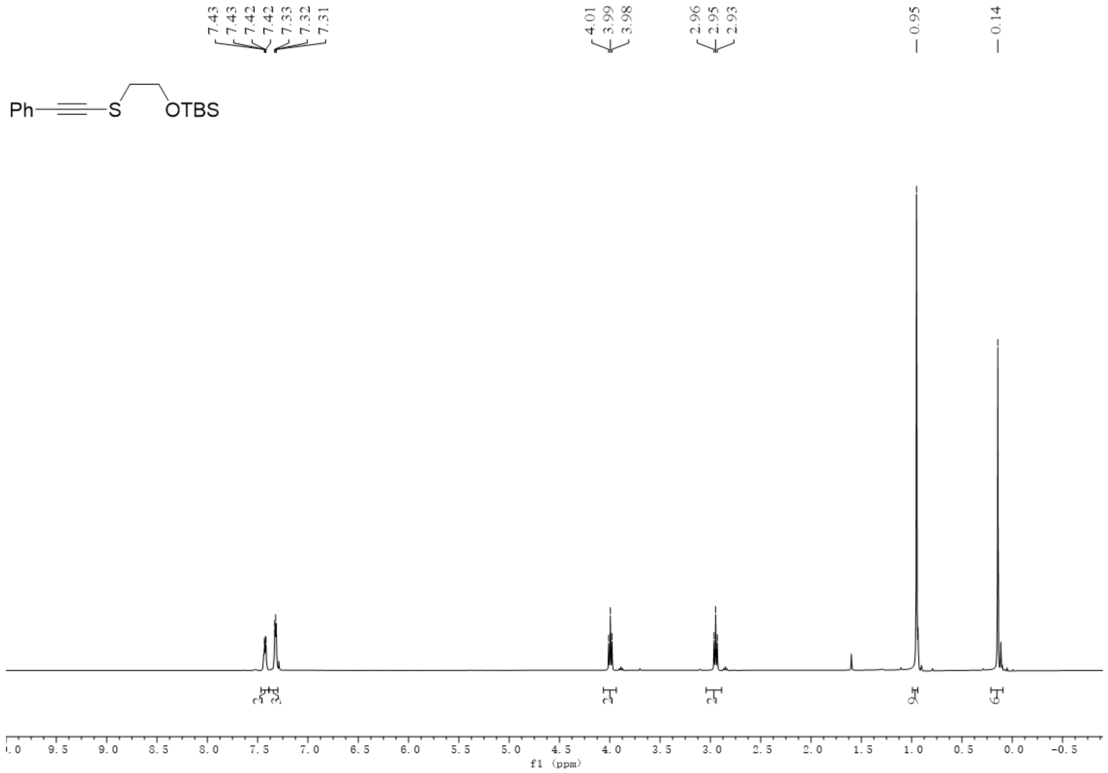

Figure S1. ^{1}H NMR spectra of **1a**.

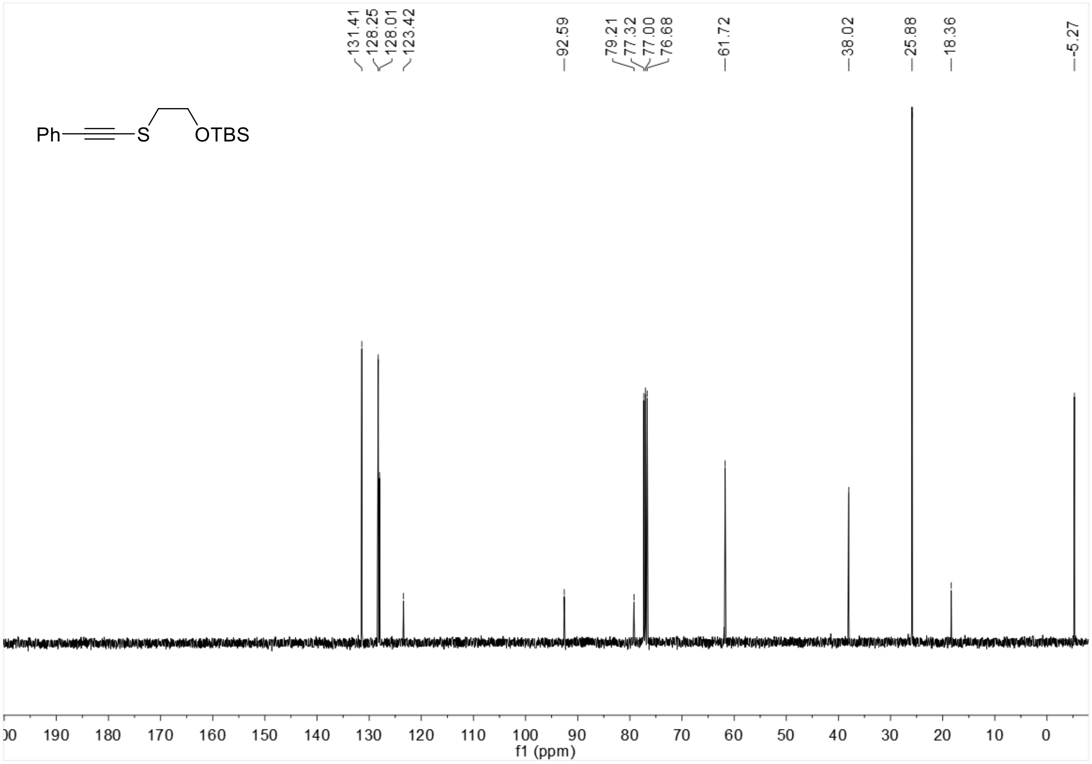

Figure S2. ^{13}C NMR spectra of **1a**.

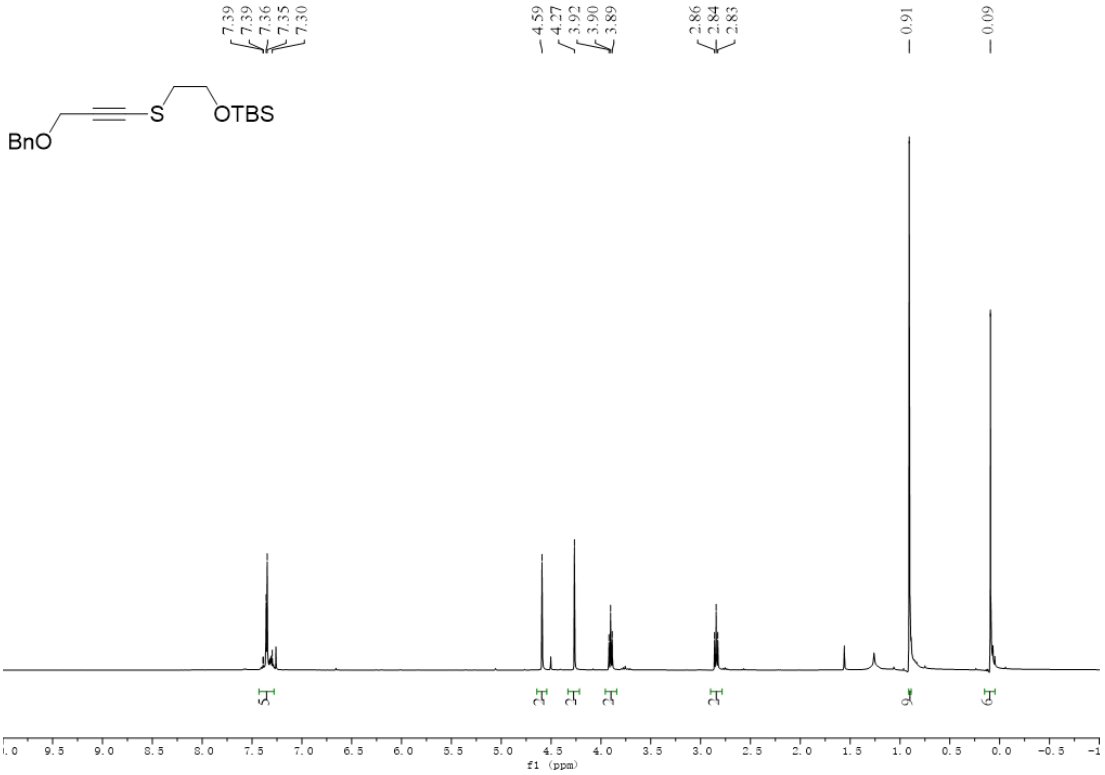

Figure S3. ^{1}H NMR spectra of **1b**.

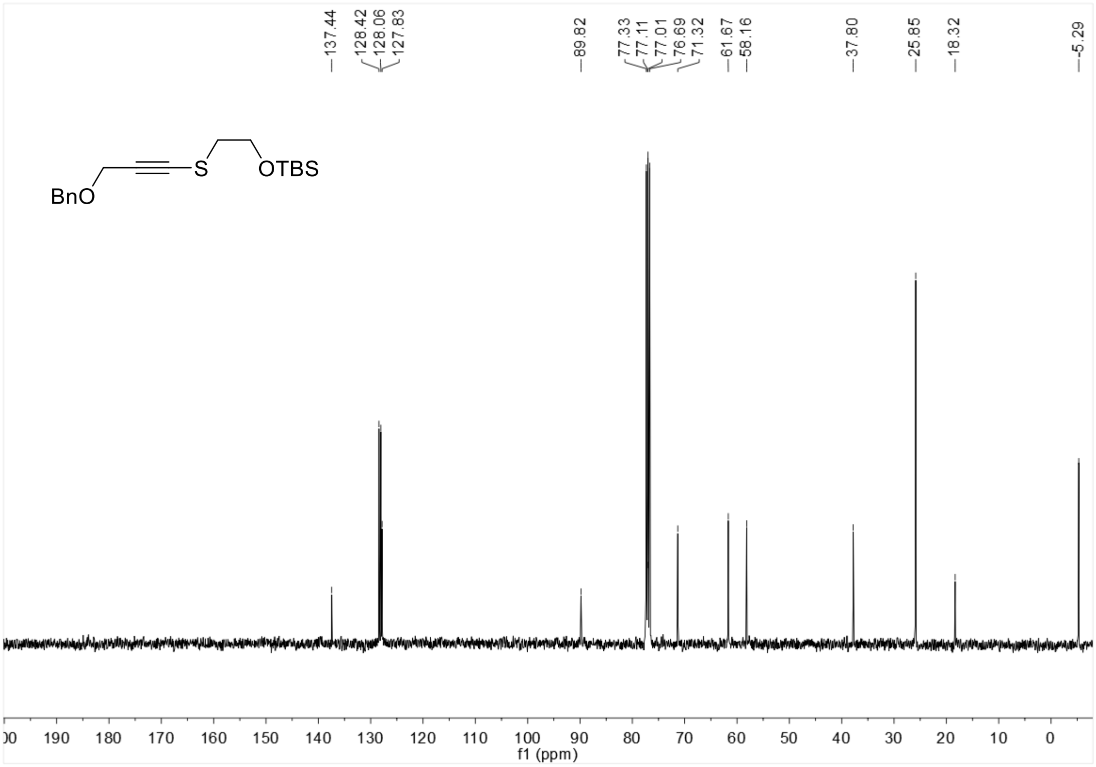

Figure S4. ^{13}C NMR spectra of **1b**.

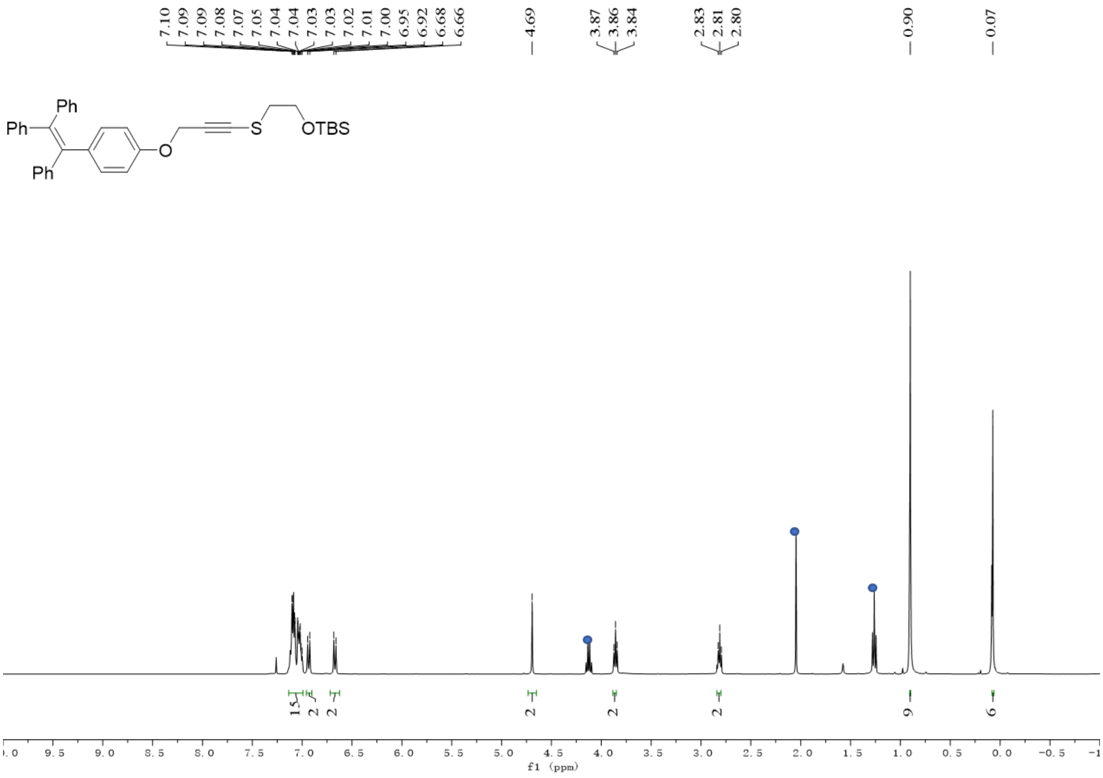

Figure S5. ^{1}H NMR spectra of **1c**.

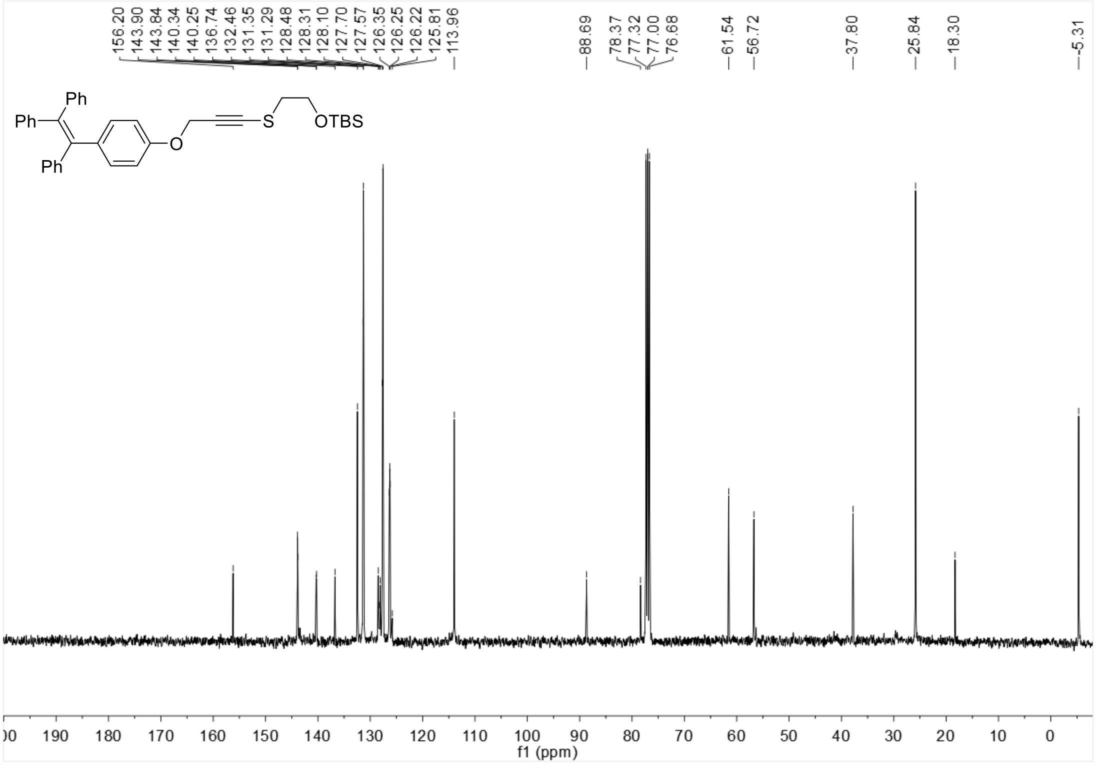

Figure S6. ^{13}C NMR spectra of **1c**.

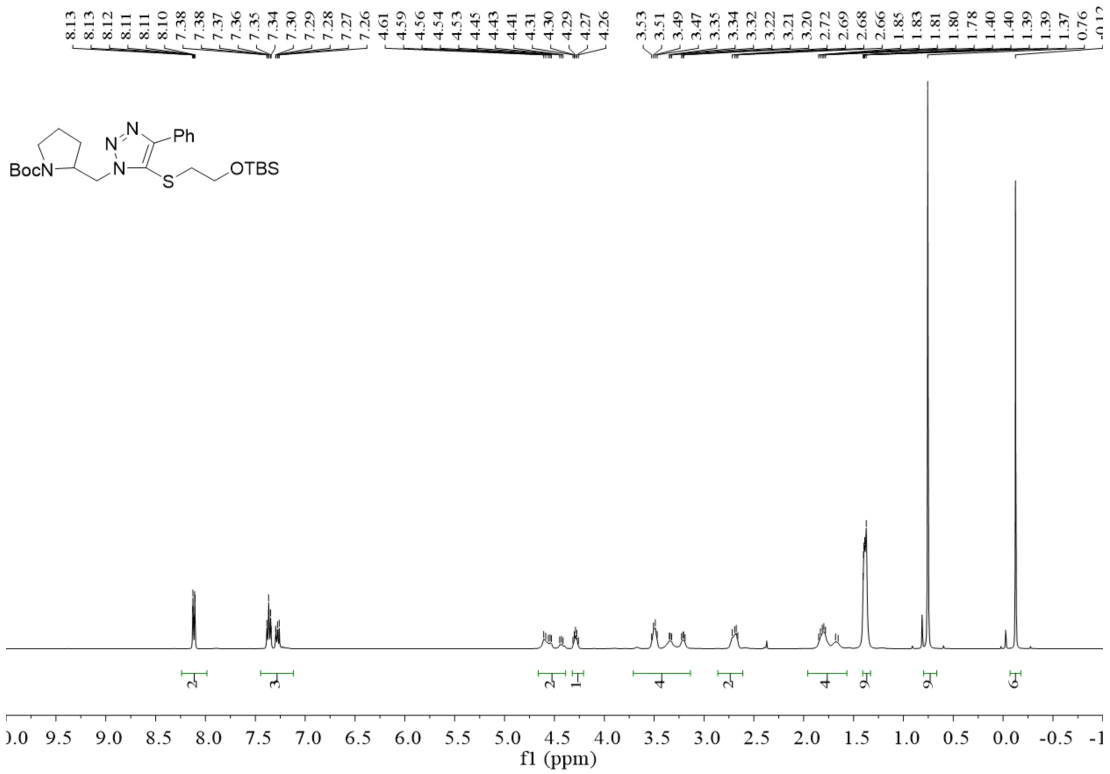

Figure S7. ^{1}H NMR spectra of **3a**.

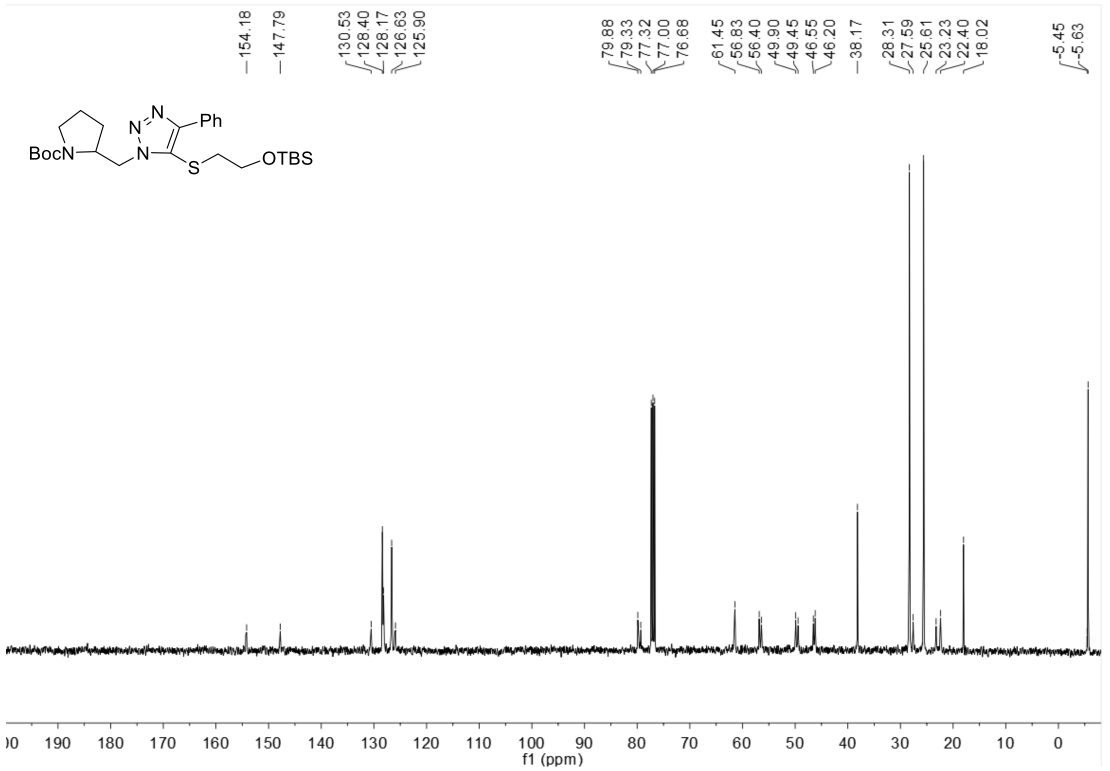

Figure S8. ^{13}C NMR spectra of **3a**.

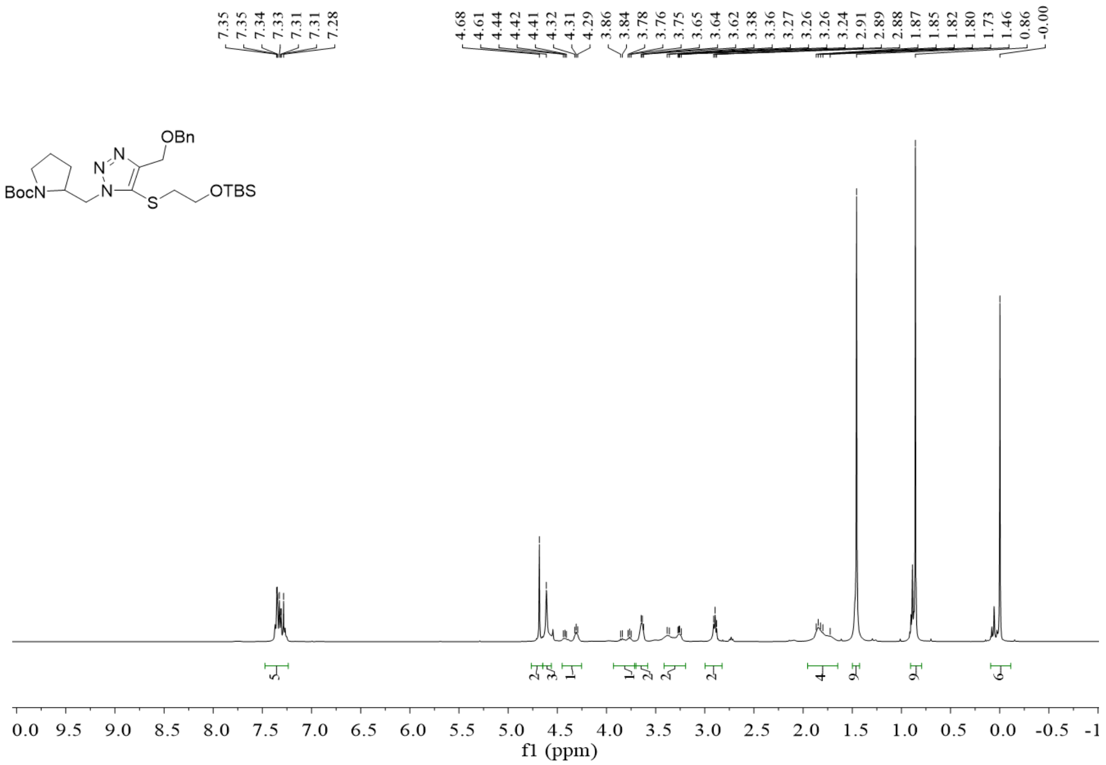

Figure S9. ^{1}H NMR spectra of **3b**.

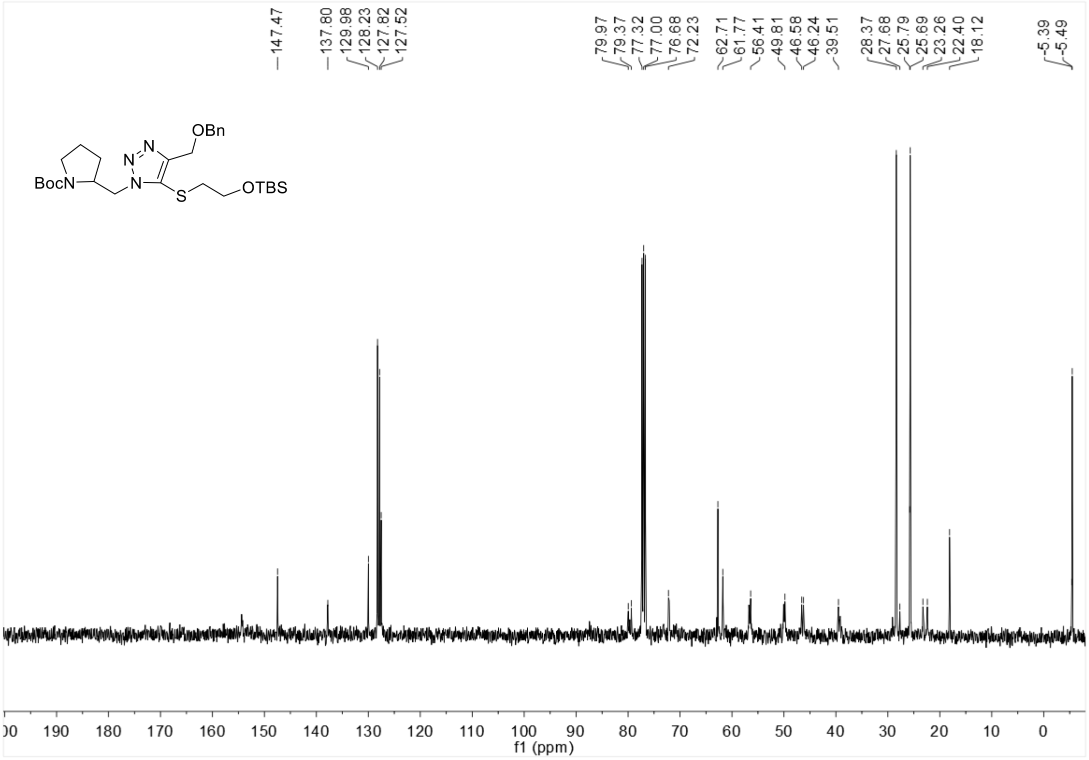

Figure S10. ^{13}C NMR spectra of **3b**.

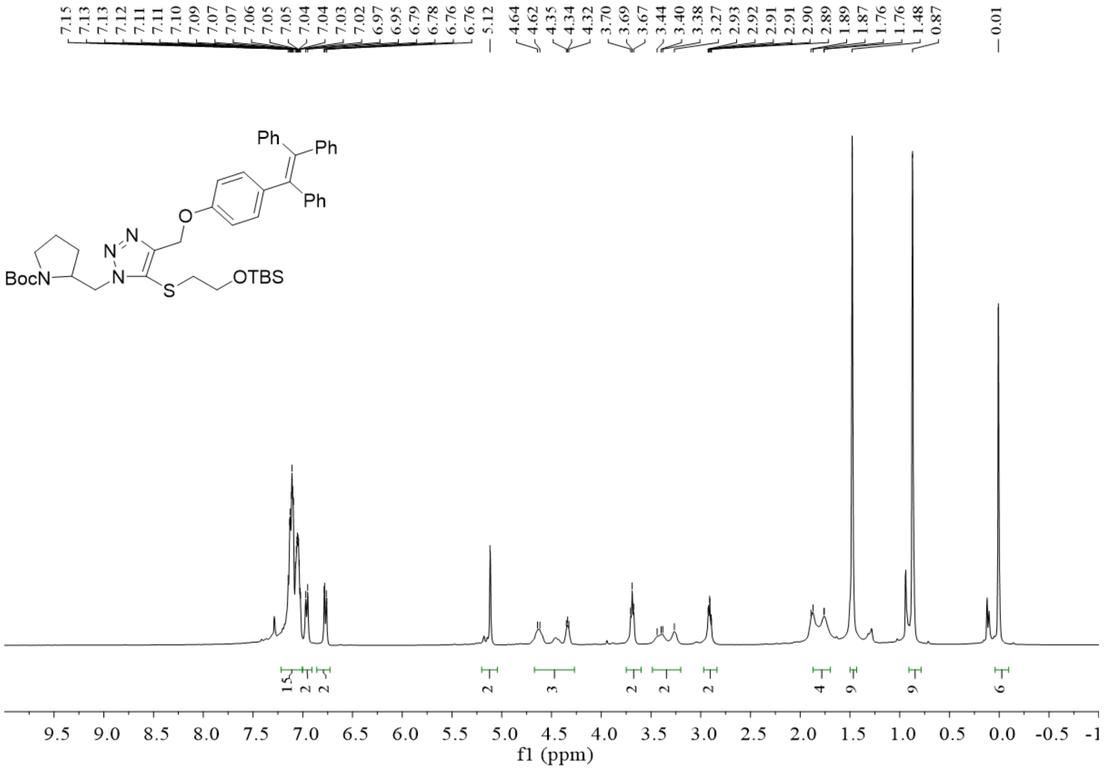

Figure S11. ^{1}H NMR spectra of **3c**.

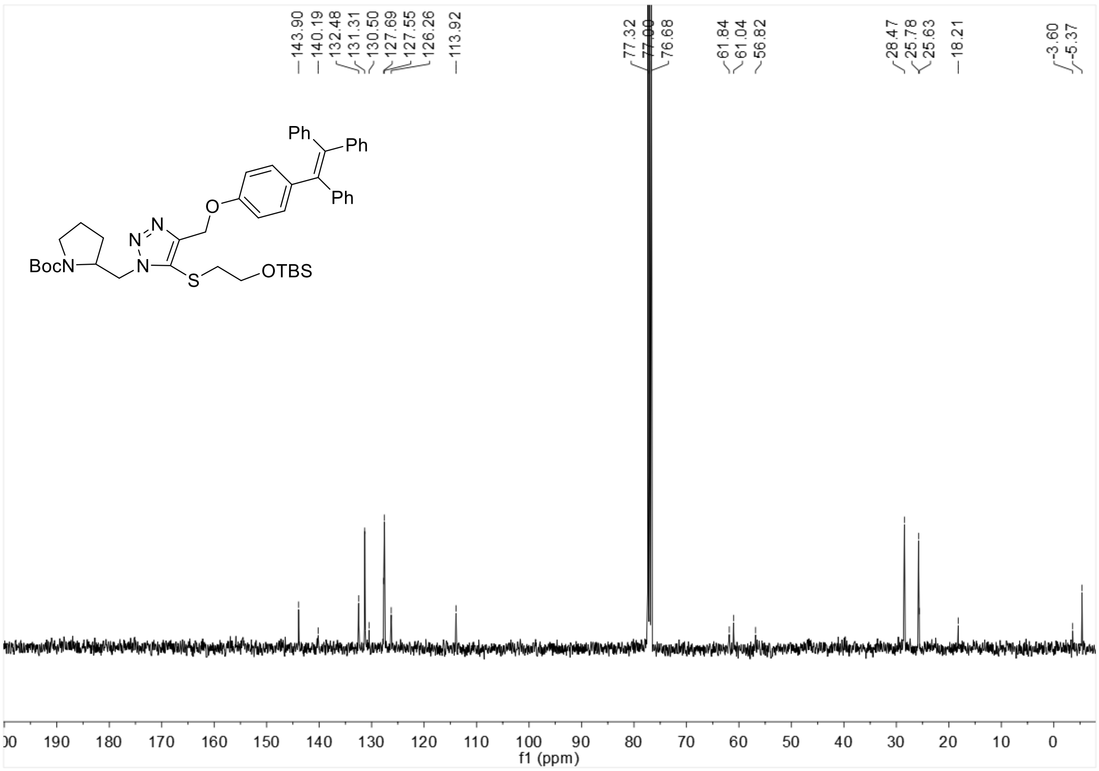

Figure S12. ^{13}C NMR spectra of **3c**.

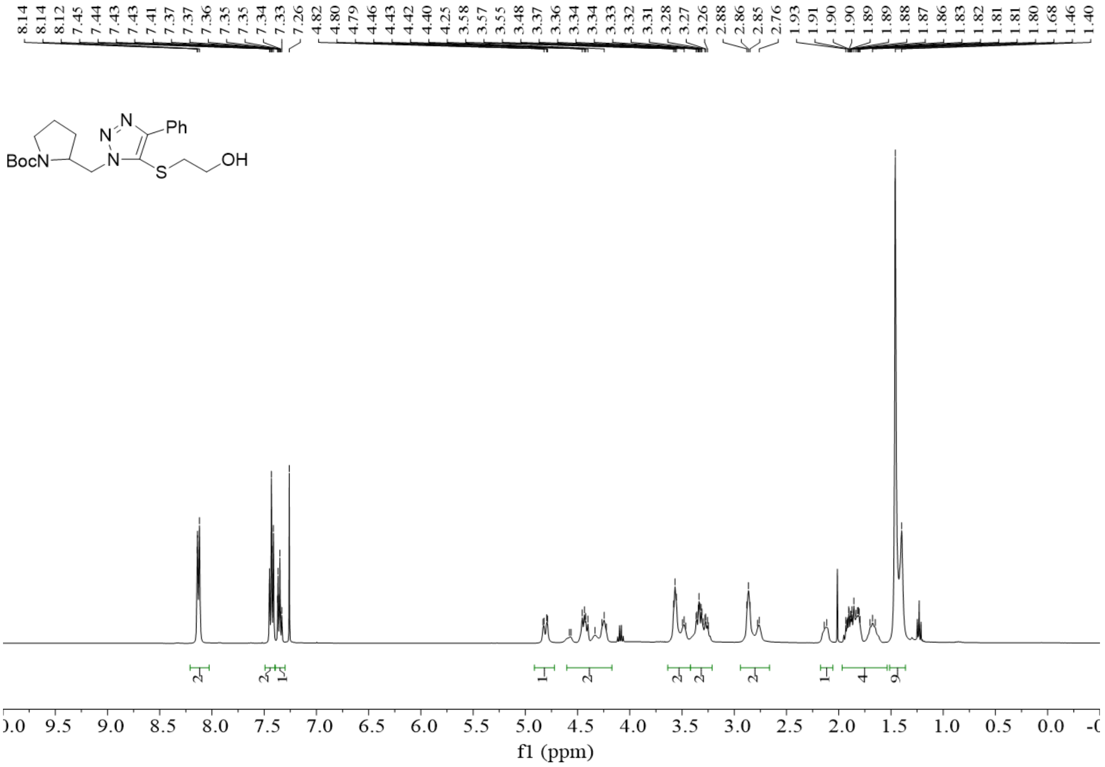

Figure S13. ^{1}H NMR spectra of **4a**.

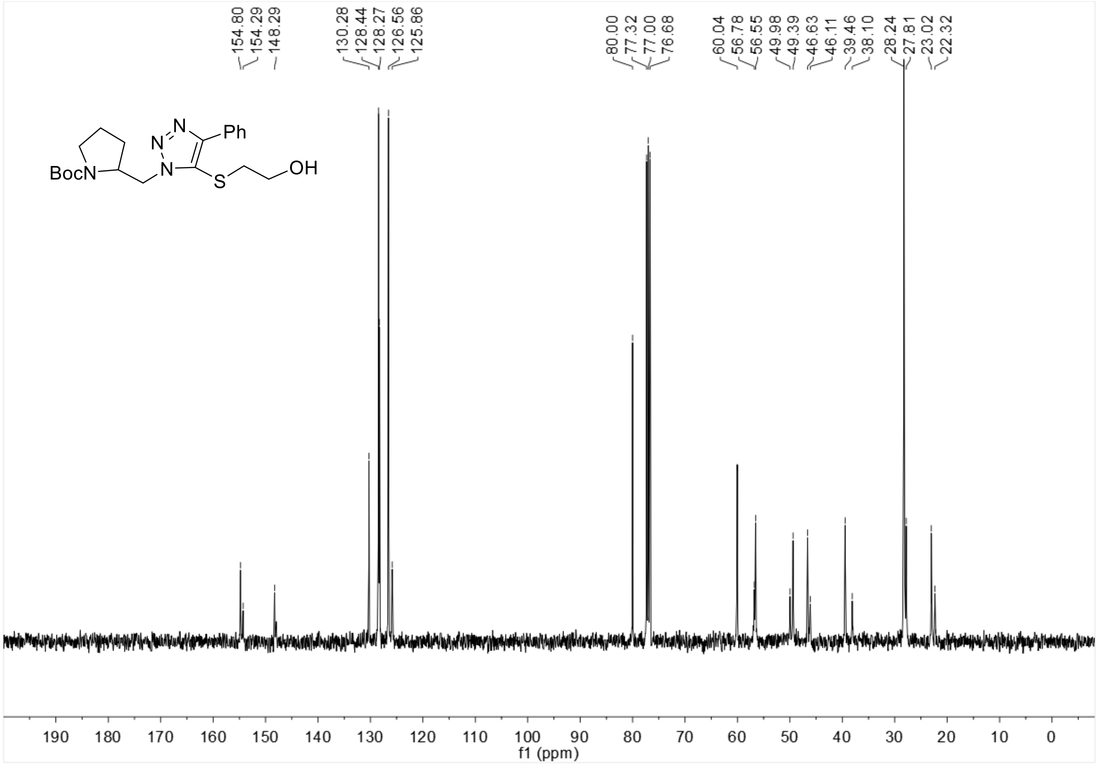

Figure S14. ^{13}C NMR spectra of **4a**.

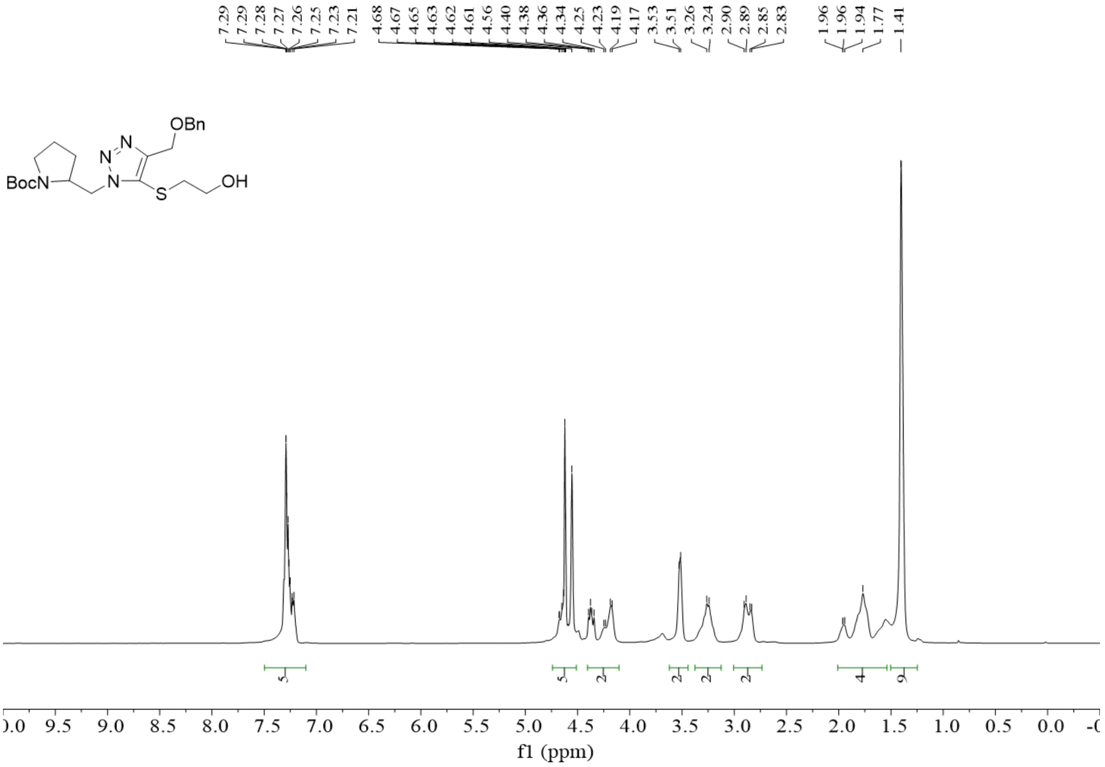

Figure S15. ^{1}H NMR spectra of **4b**.

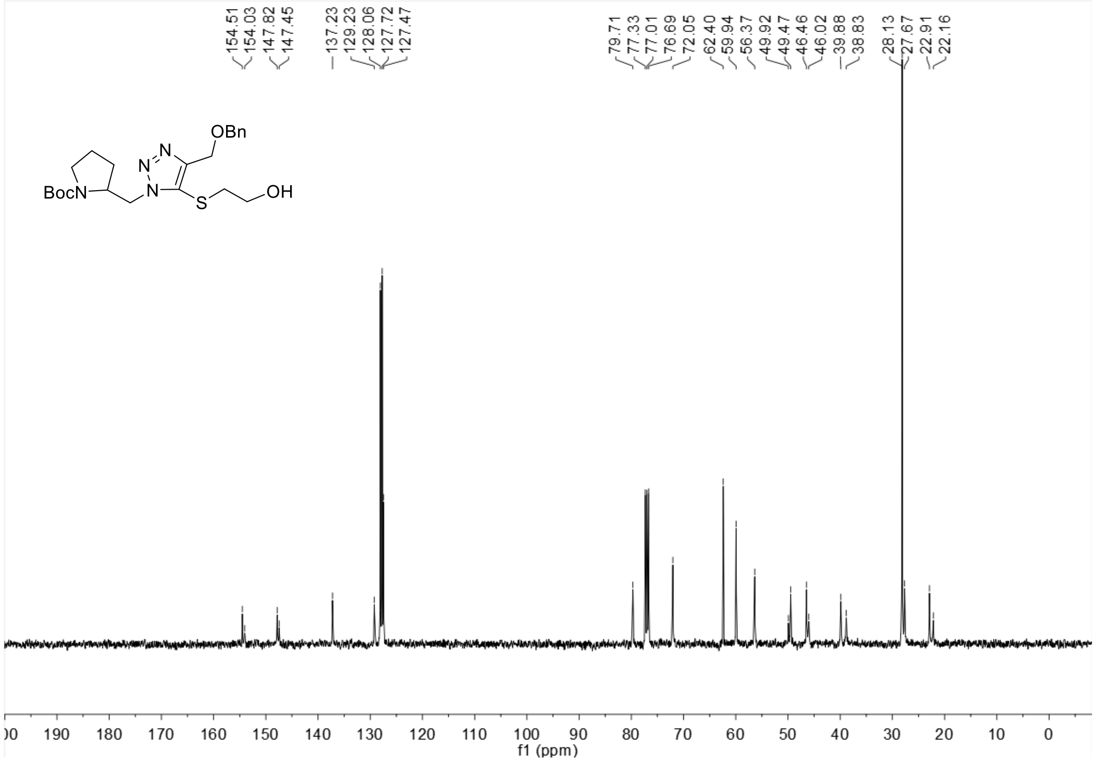

Figure S16. ^{13}C NMR spectra of **4b**.

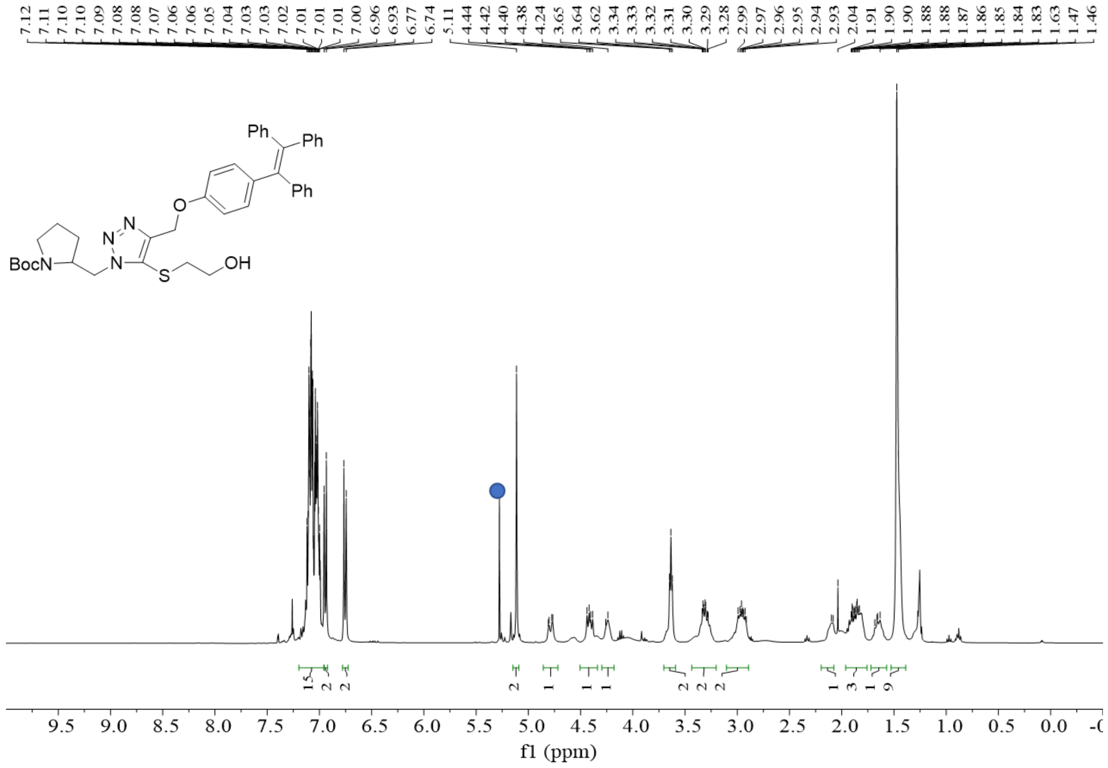

Figure S17. ^{1}H NMR spectra of **4c**.

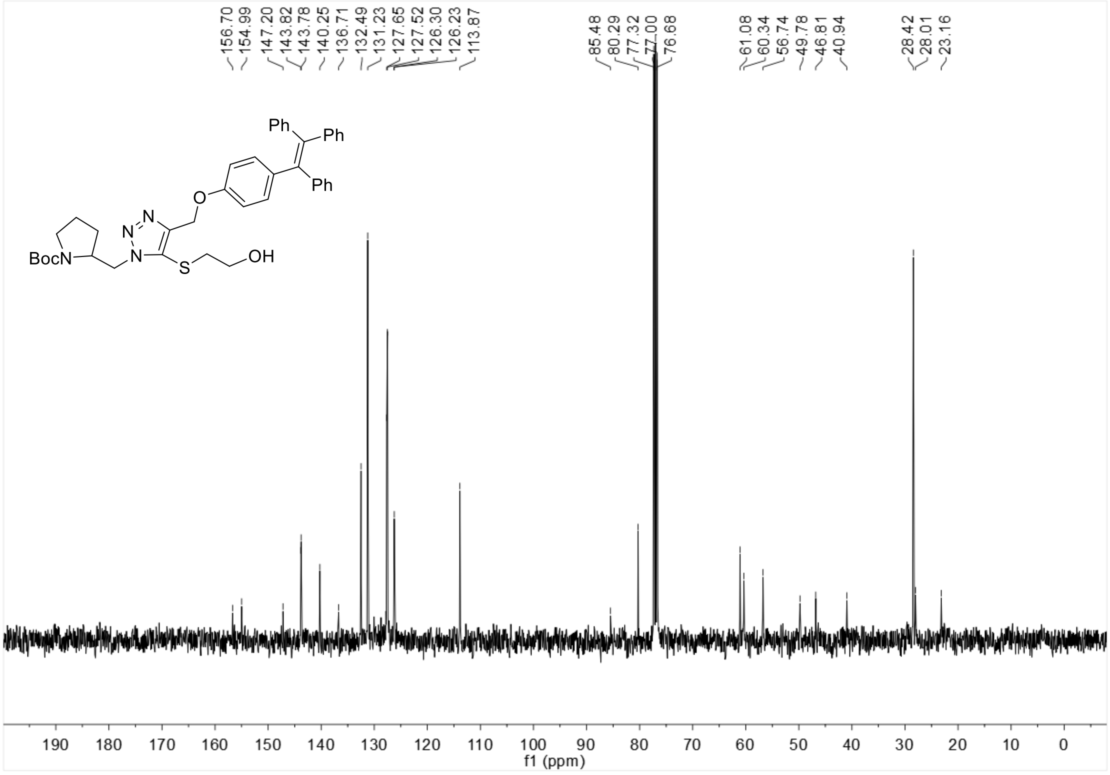

Figure S18. ^{13}C NMR spectra of **4c**.

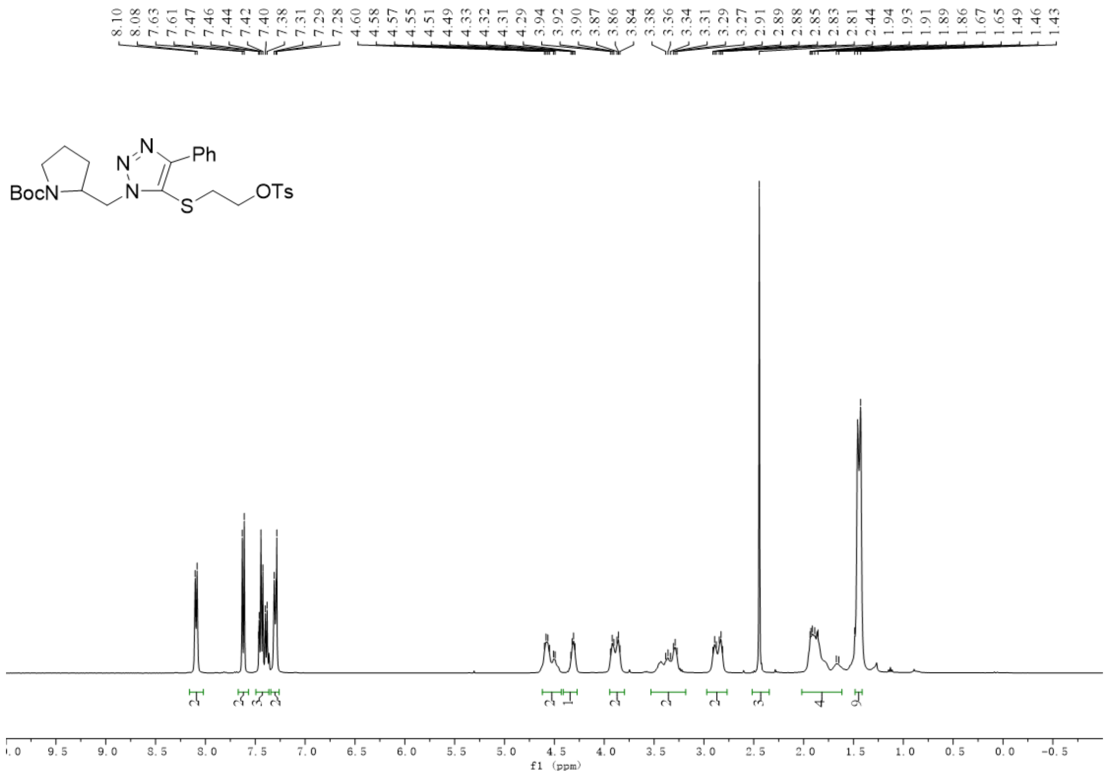

Figure S19. ^{1}H NMR spectra of **4a**-OTs.

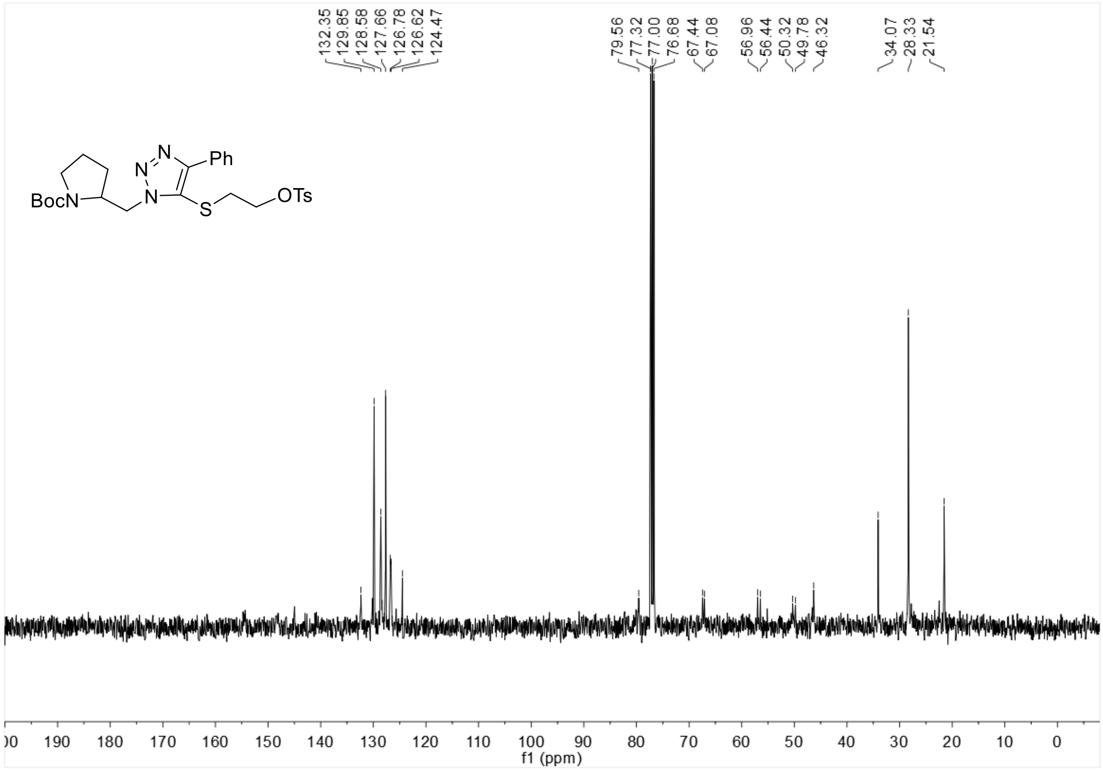

Figure S20. ^{13}C NMR spectra of **4a**-OTs.

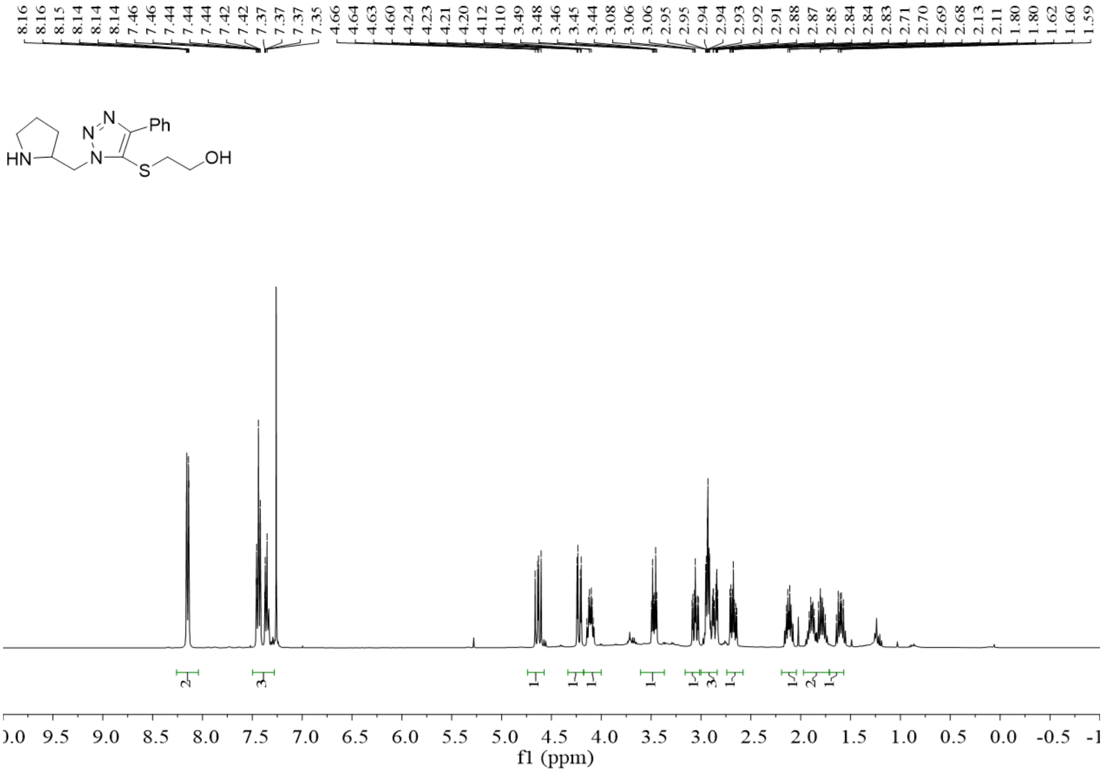

Figure S21. ^{1}H NMR spectra of **4a**-H.

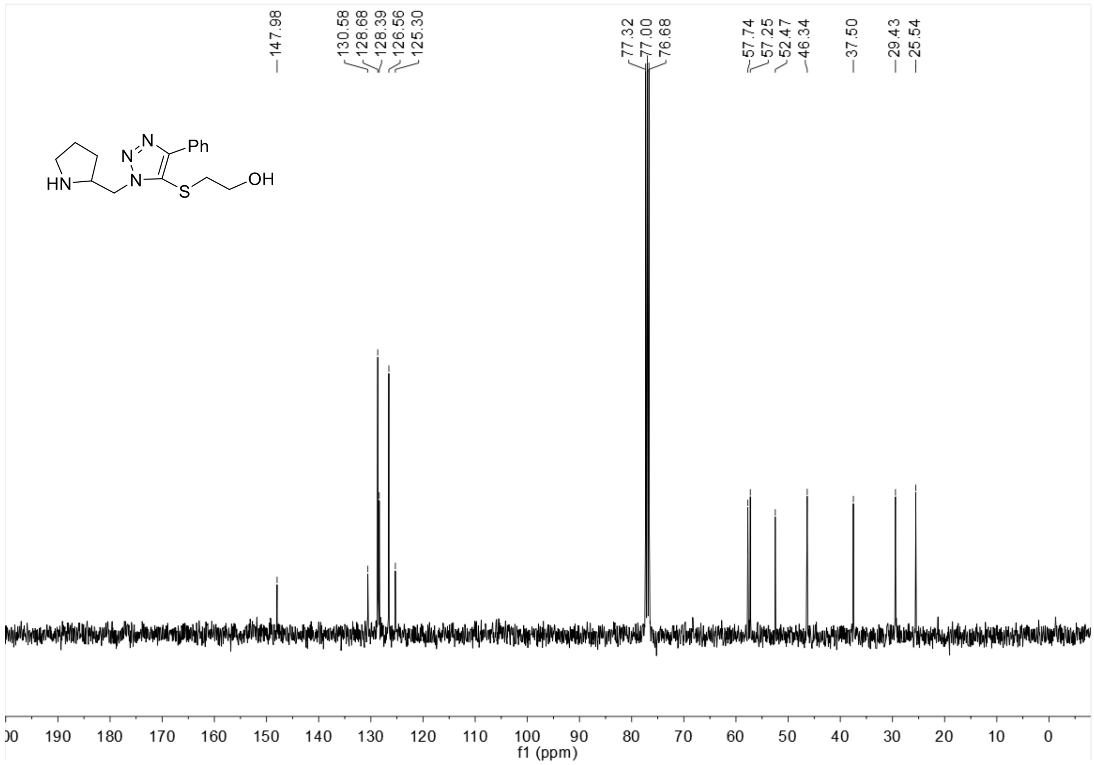

Figure S22. ^{13}C NMR spectra of **4a**-H.

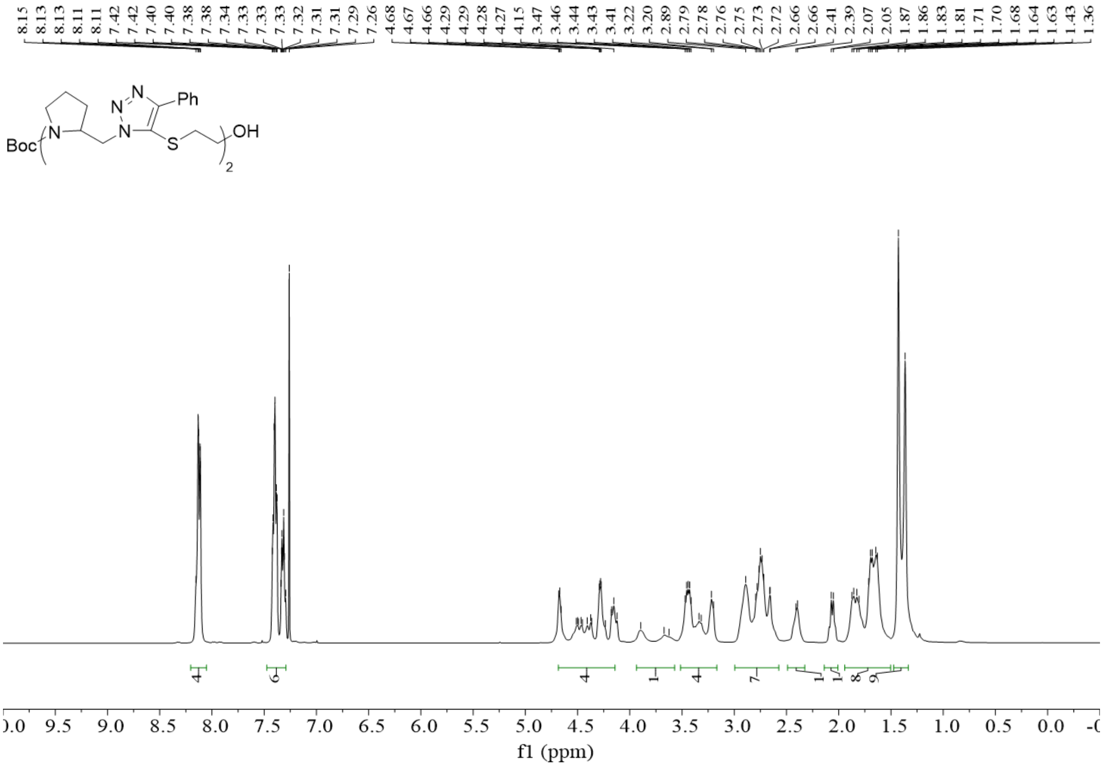

Figure S23. ^{1}H NMR spectra of **5a**.

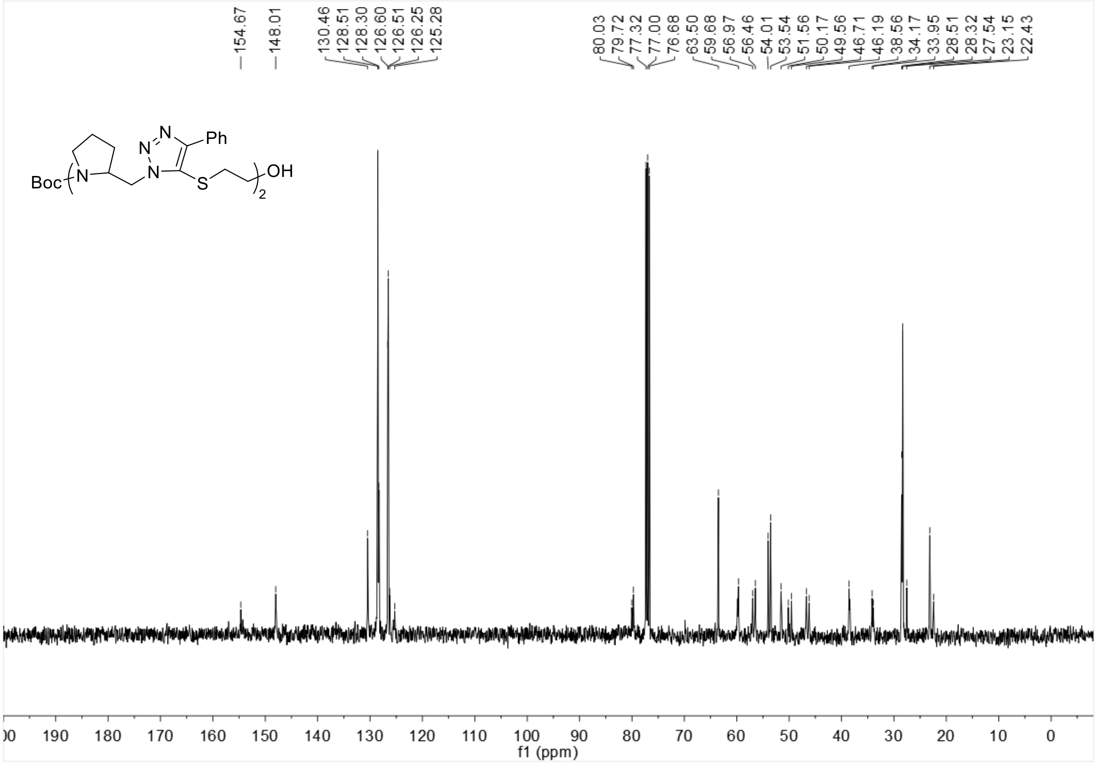

Figure S24. ^{13}C NMR spectra of **5a**.

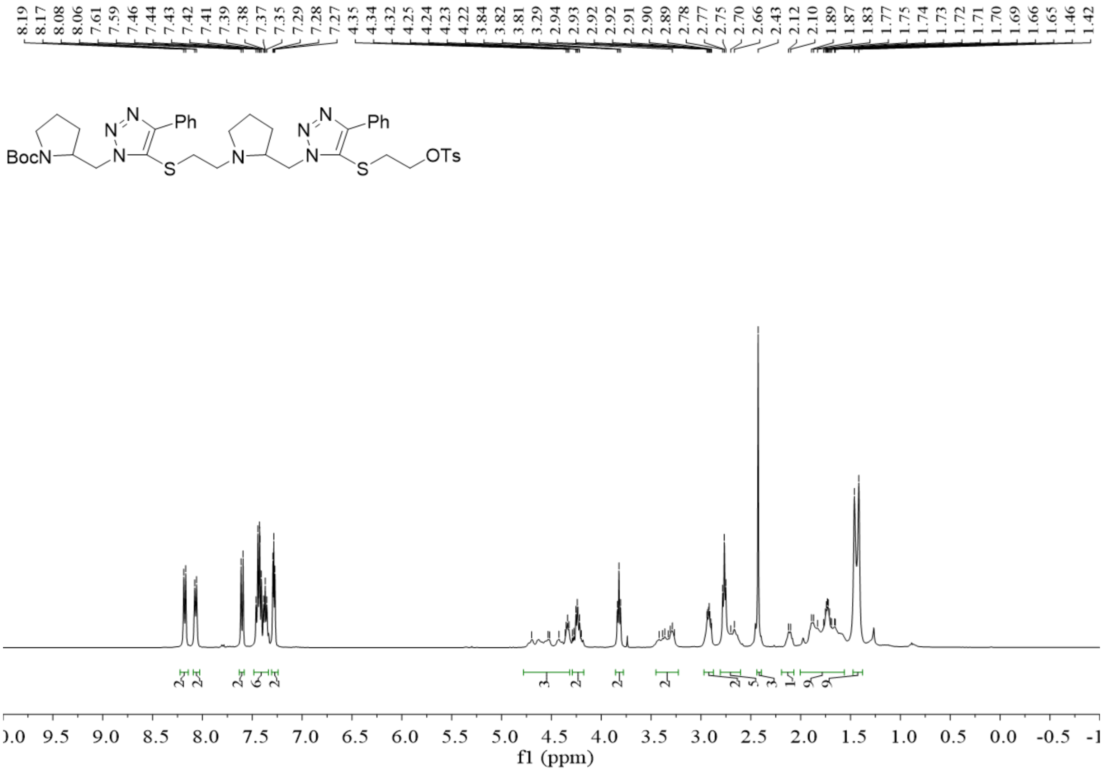

Figure S25. ^{1}H NMR spectra of **5a**-OTs.

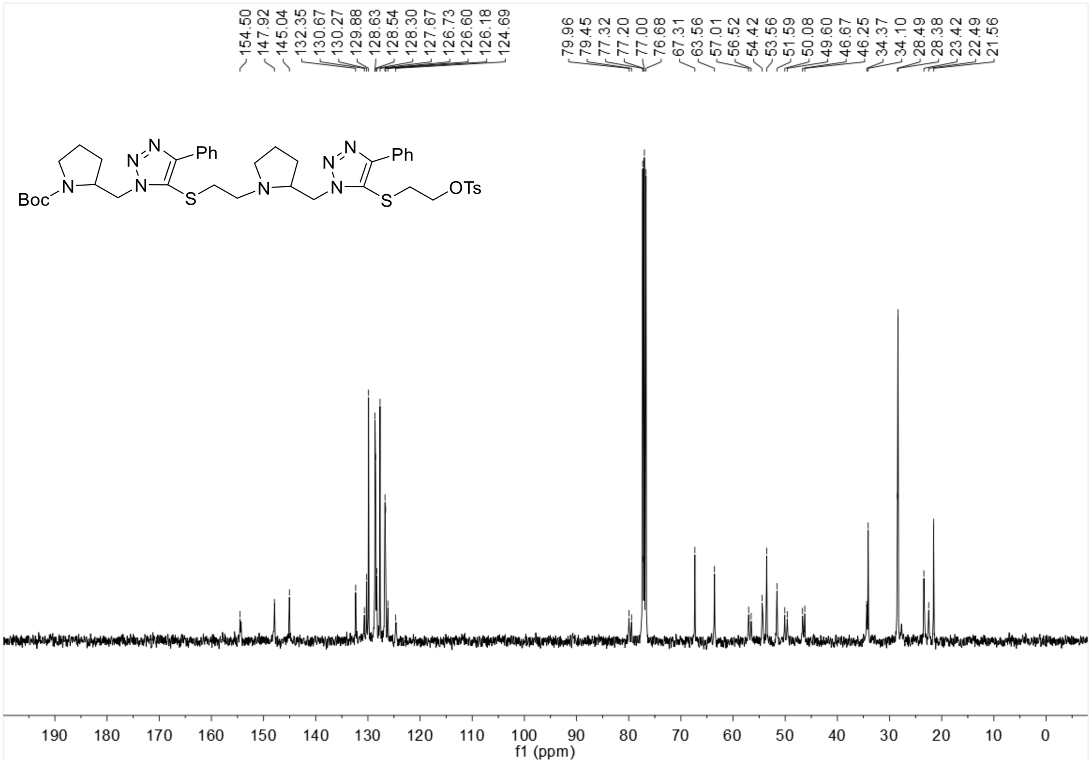

Figure S26. ^{13}C NMR spectra of **5a**-OTs.

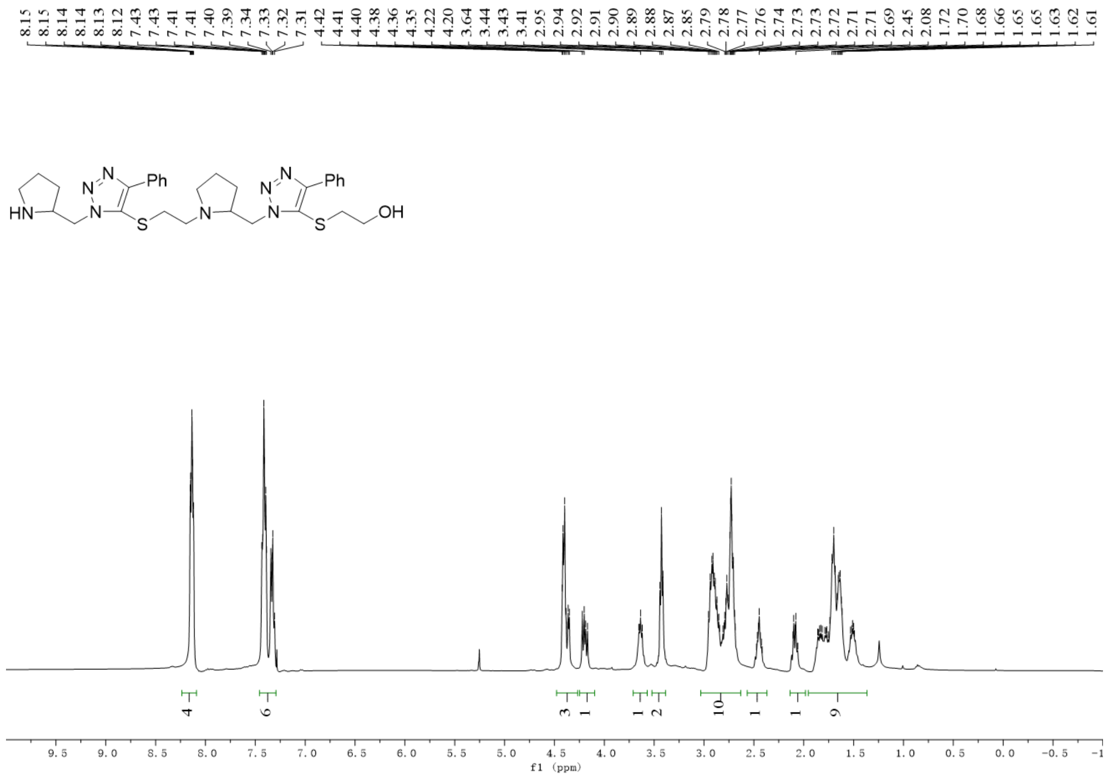

Figure S27. ^{1}H NMR spectra of **5a**-H.

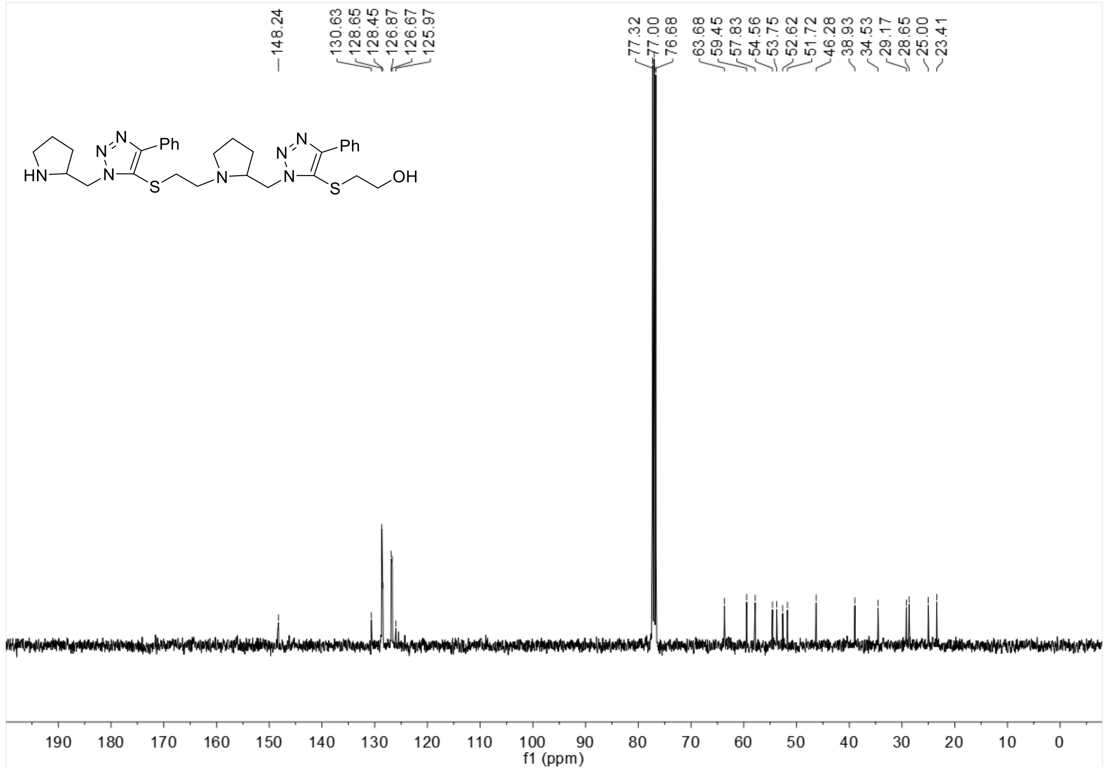

Figure S28. ^{13}C NMR spectra of **5a**-H.

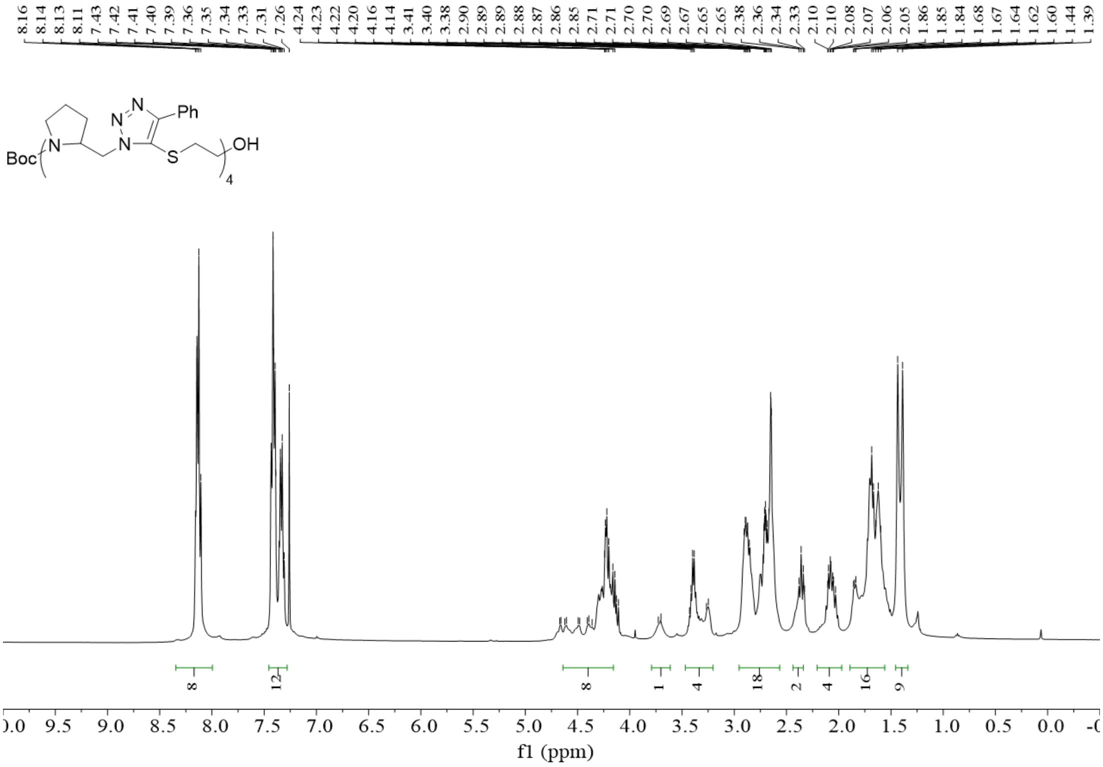

Figure S29. ^{1}H NMR spectra of **6a**.

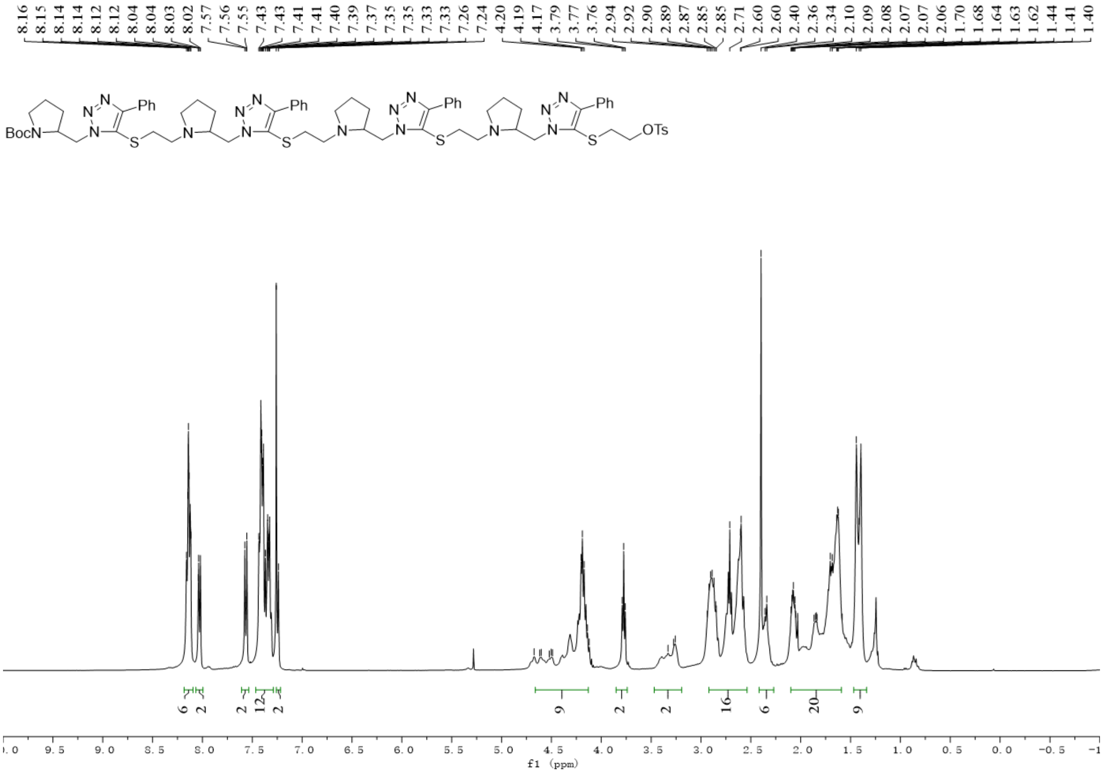

Figure S30. ^{1}H NMR spectra of **6a**-OTs.

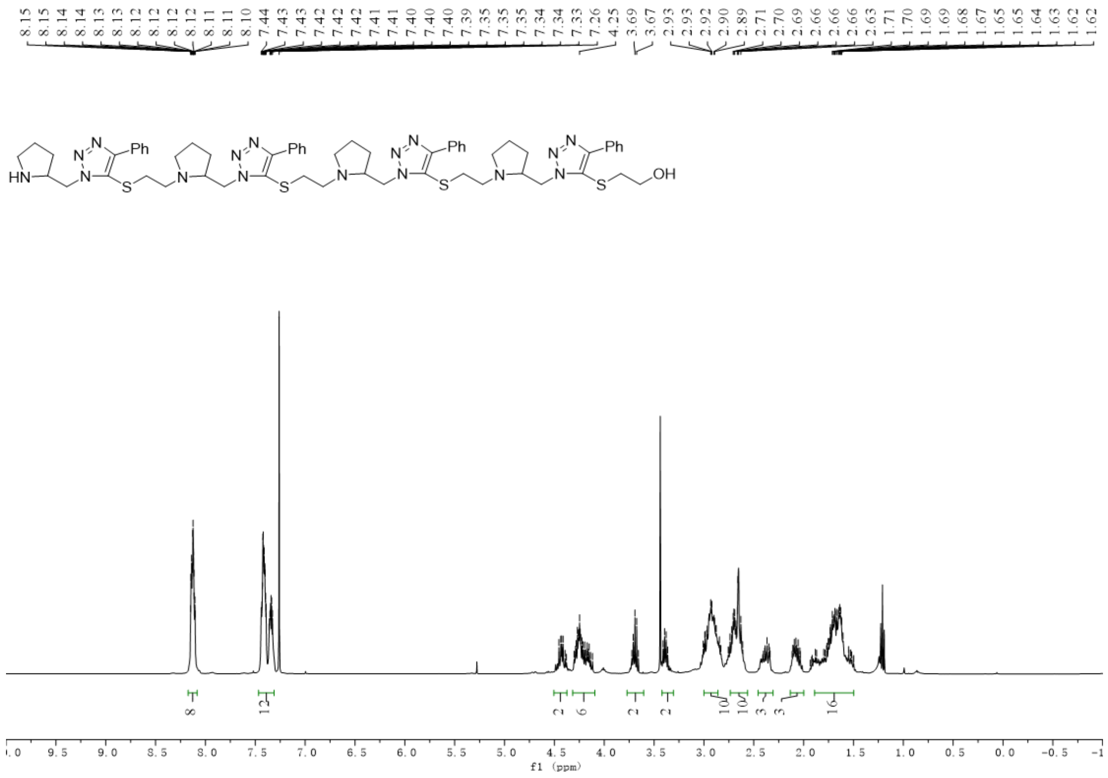

Figure S31. ^{1}H NMR spectra of **6a**-H.

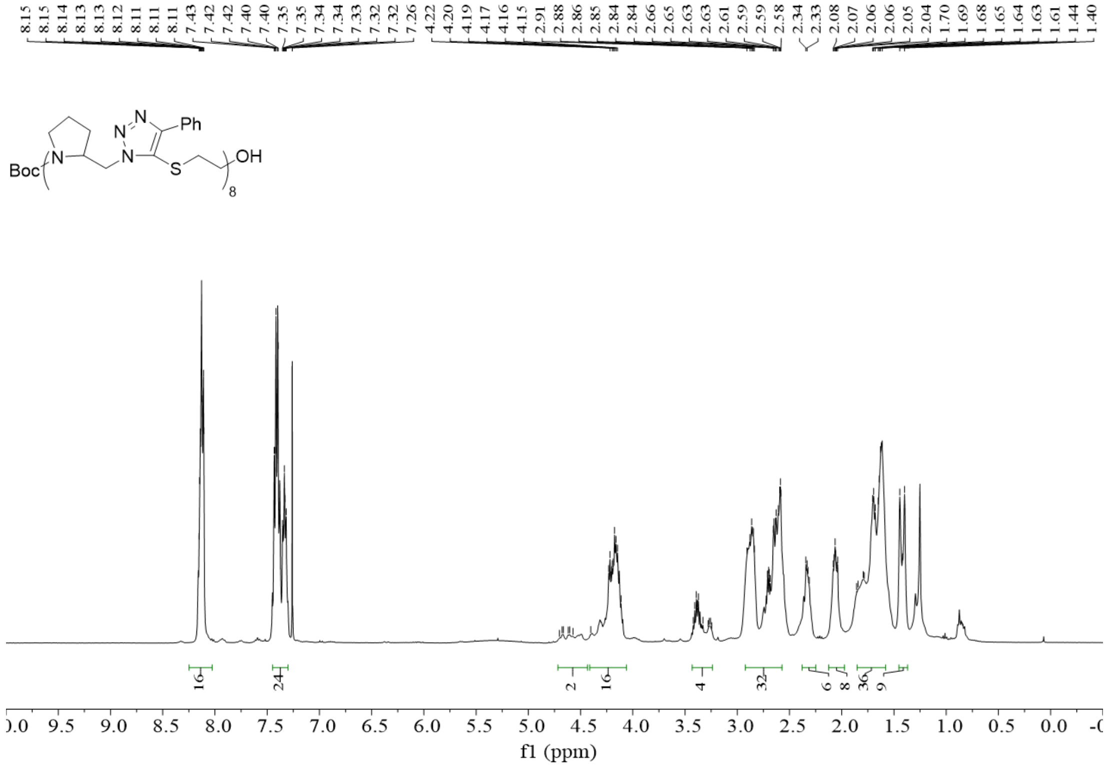

Figure S32. ^{1}H NMR spectra of **8a**.

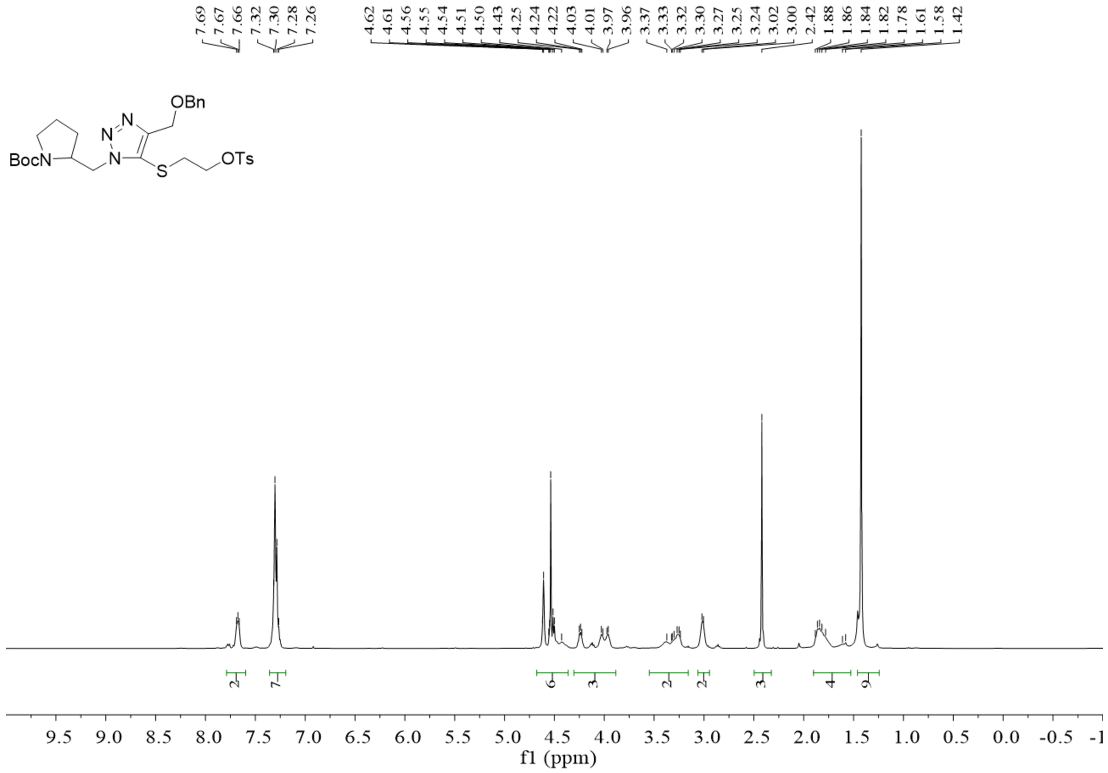

Figure S33. ^{1}H NMR spectra of **4b**-OTs.

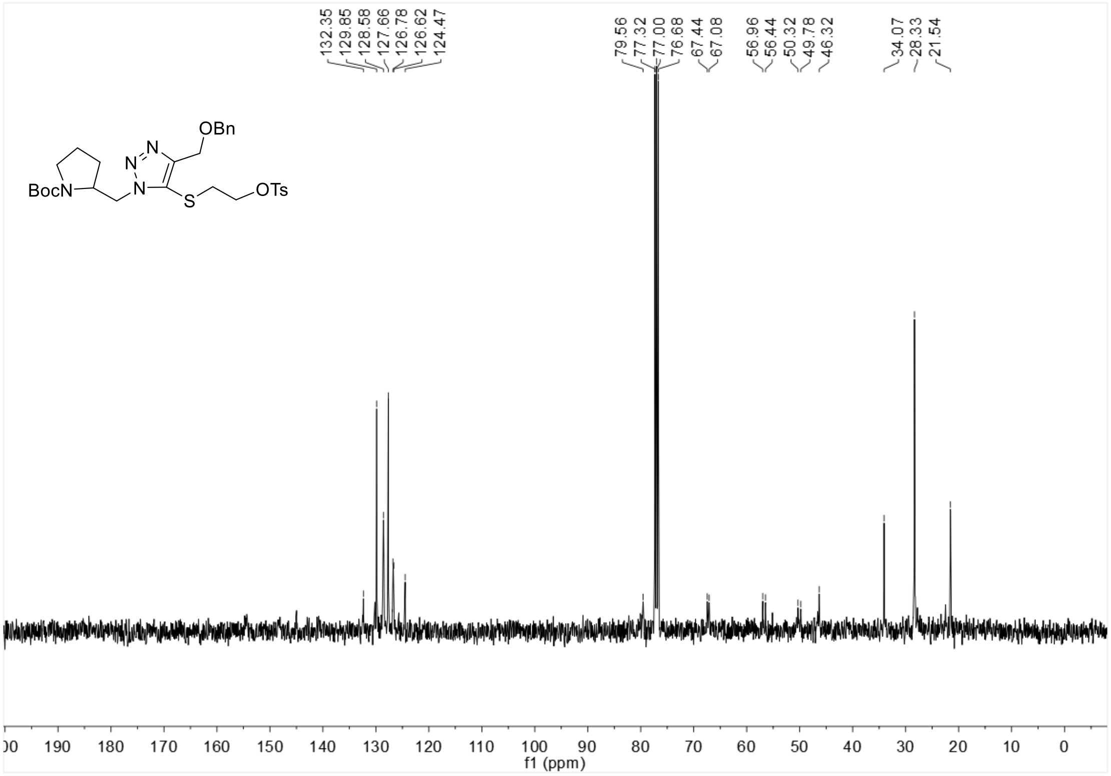

Figure S34. ^{13}C NMR spectra of **4b**-OTs.

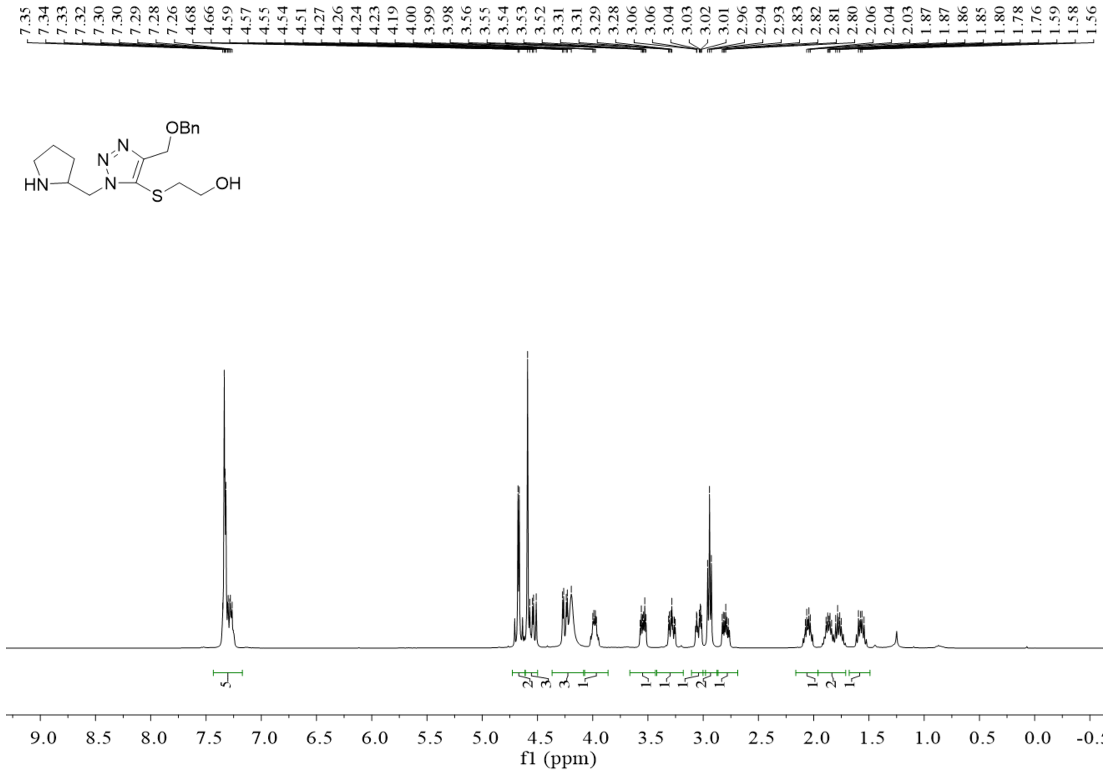

Figure S35. ^{1}H NMR spectra of **4b**-H.

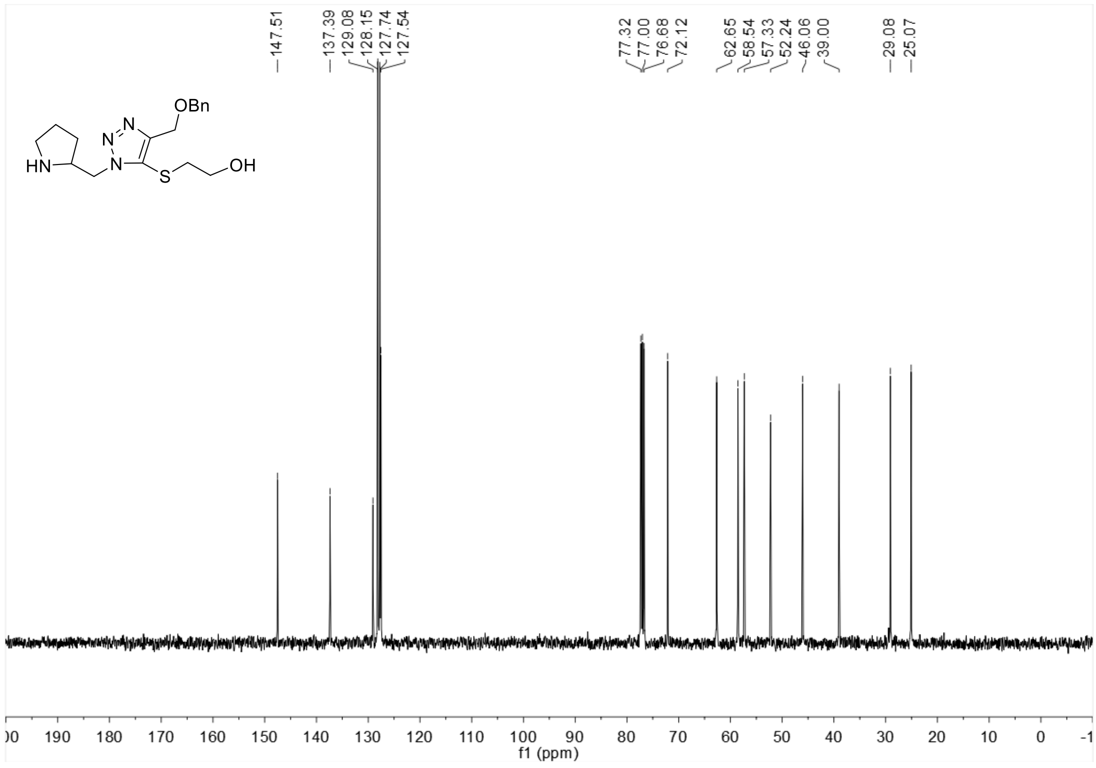

Figure S36. ^{13}C NMR spectra of **4b**-H.

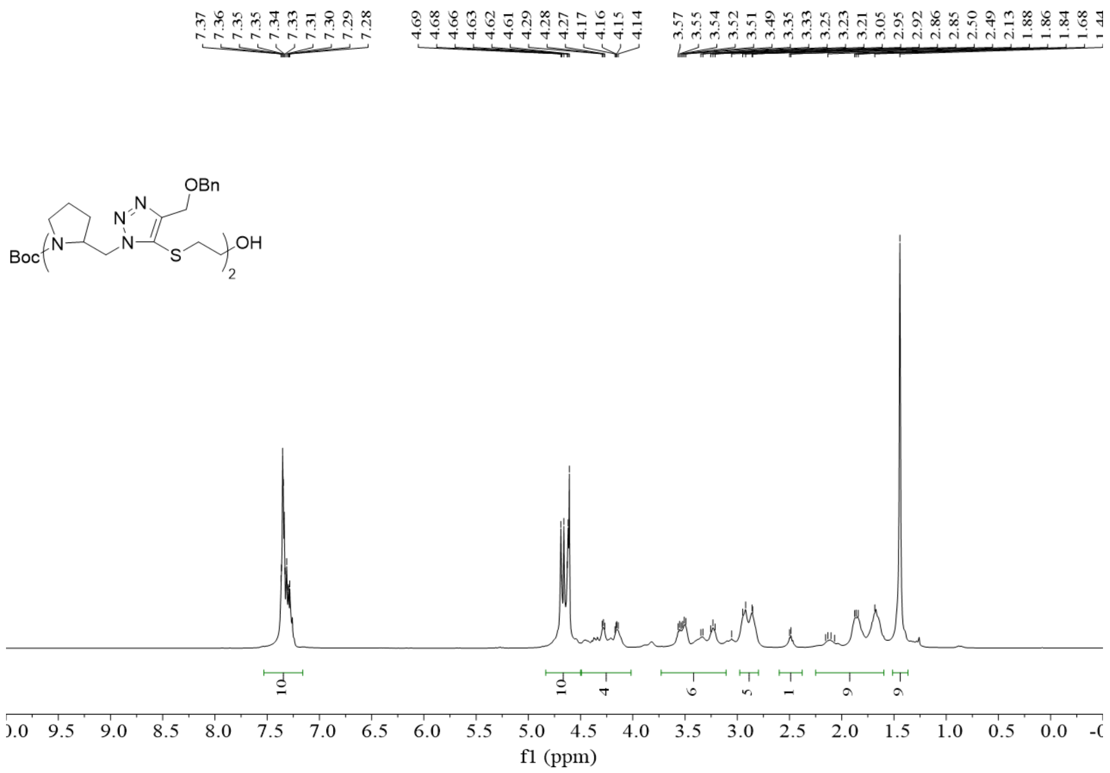

Figure S37. ^{1}H NMR spectra of **5b**.

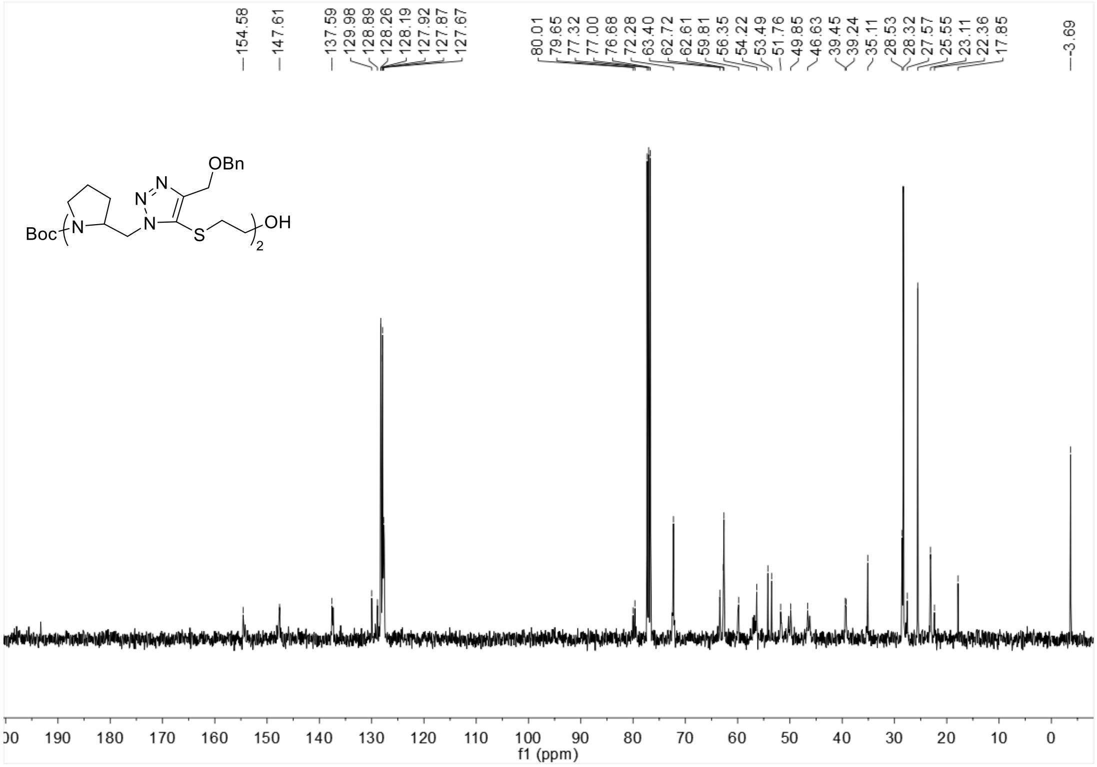

Figure S38. ^{13}C NMR spectra of **5b**.

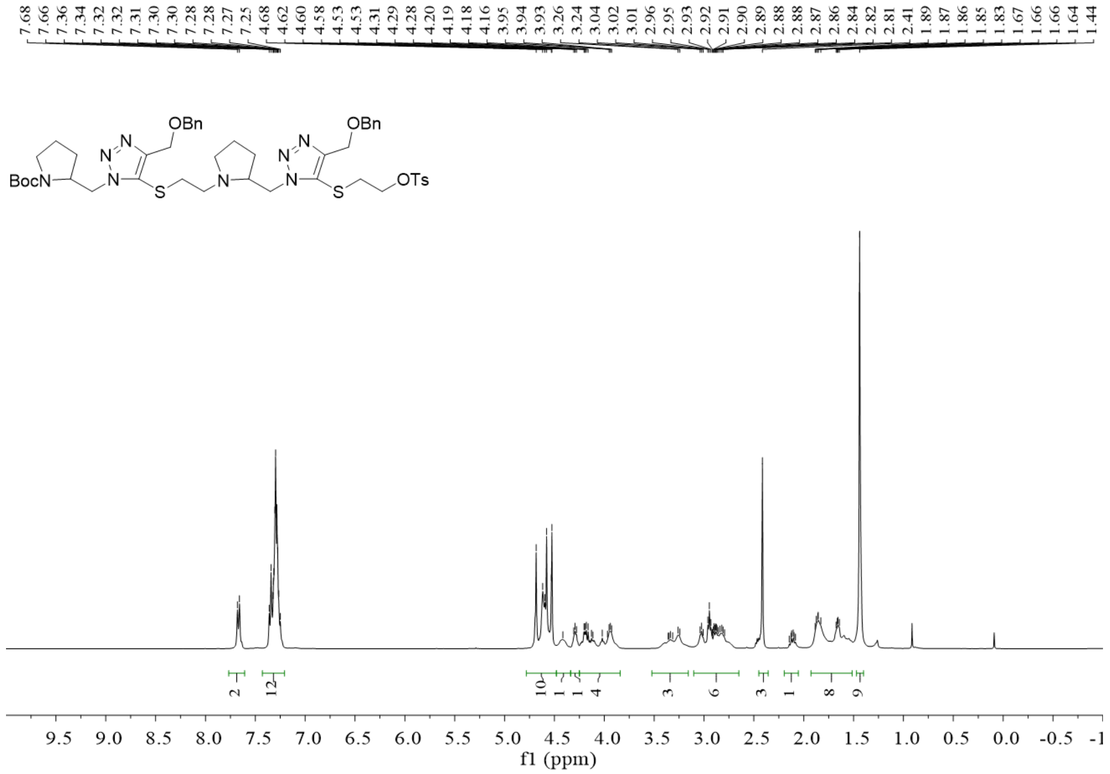

Figure S39. ^{1}H NMR spectra of **5b**-OTs.

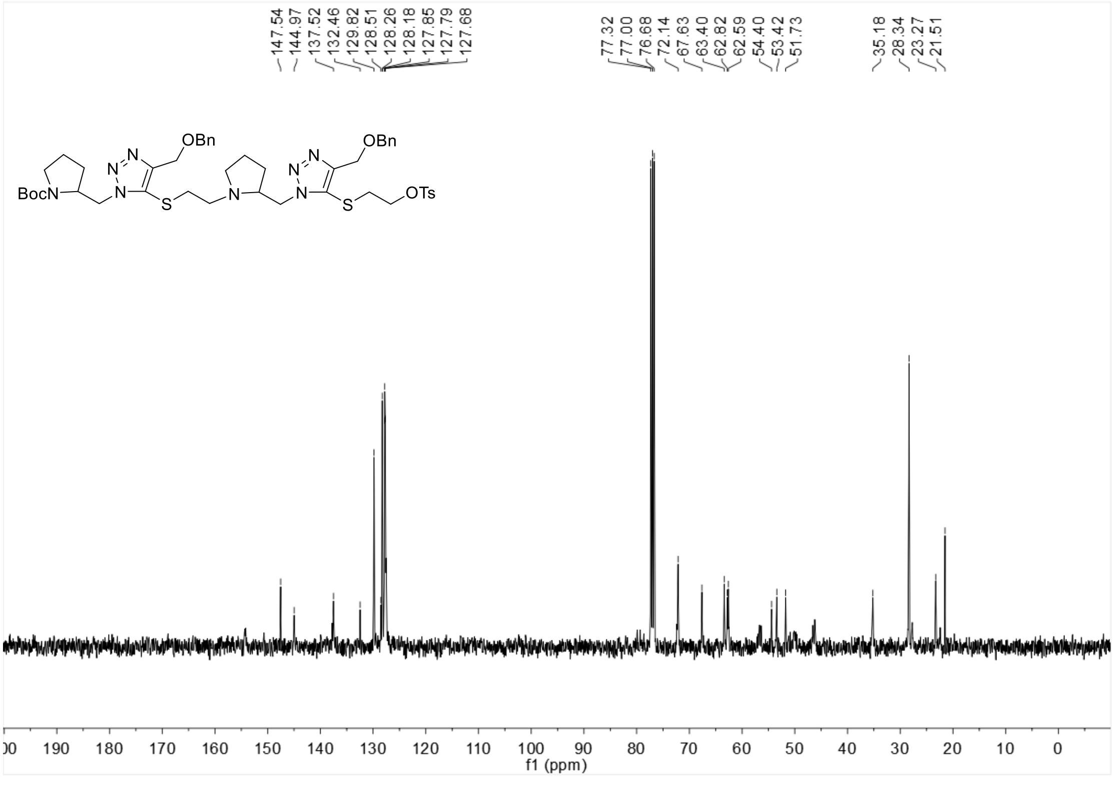

Figure S40. ^{13}C NMR spectra of **5b**-OTs.

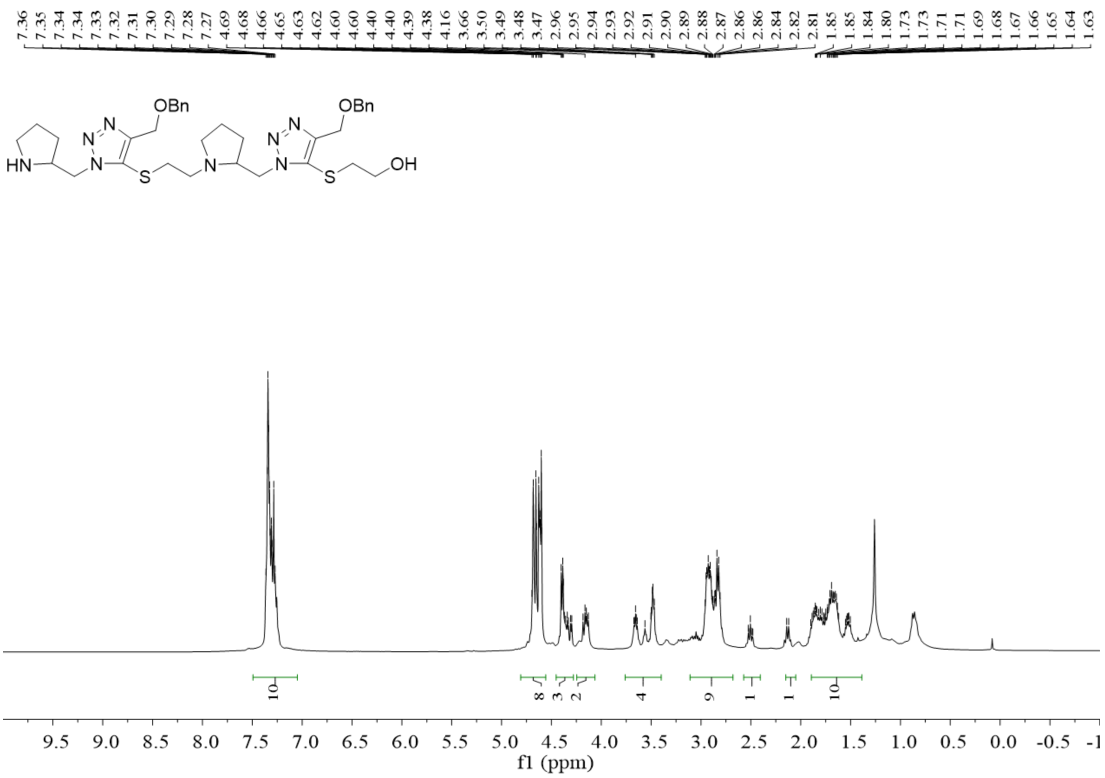

Figure S41. ^{1}H NMR spectra of **5b**-H.

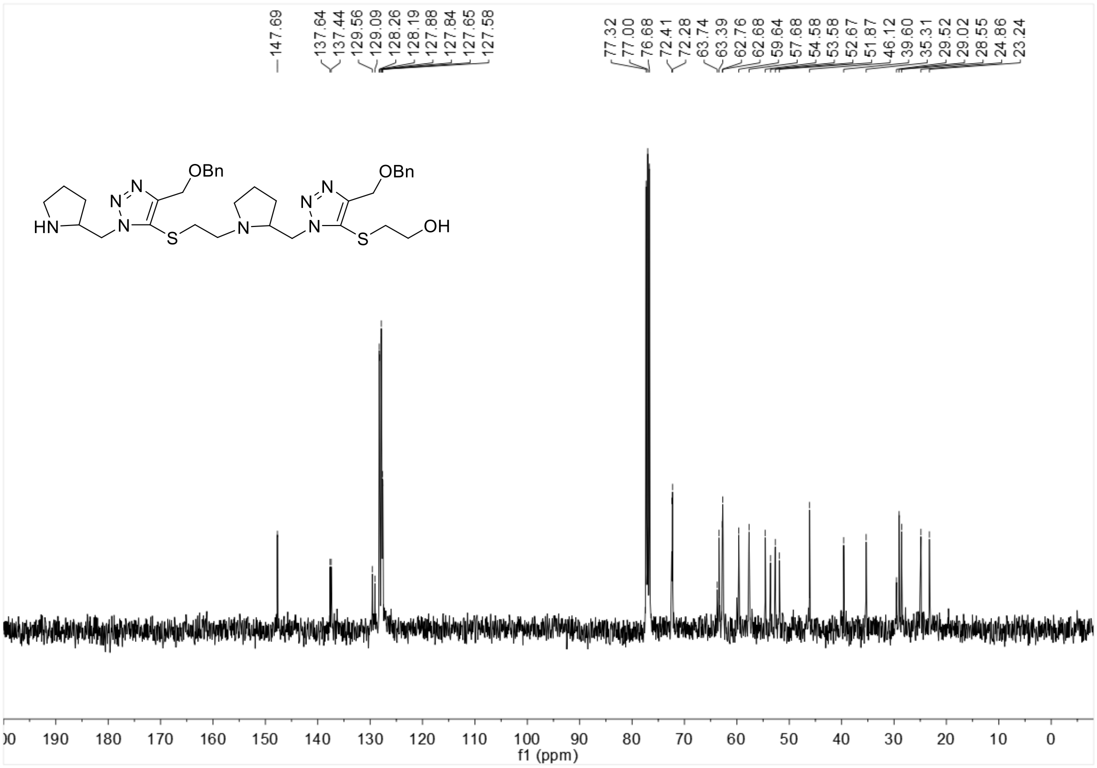

Figure S42. ^{13}C NMR spectra of **5b**-H.

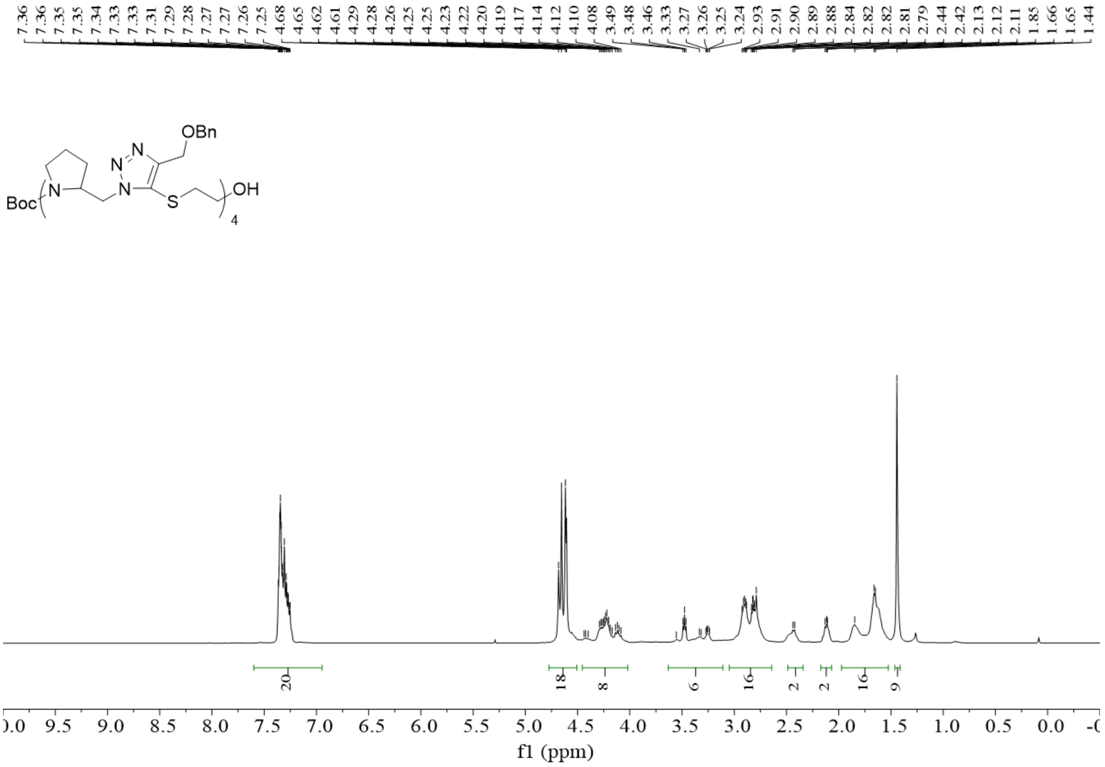

Figure S43. ^{1}H NMR spectra of **6b**.

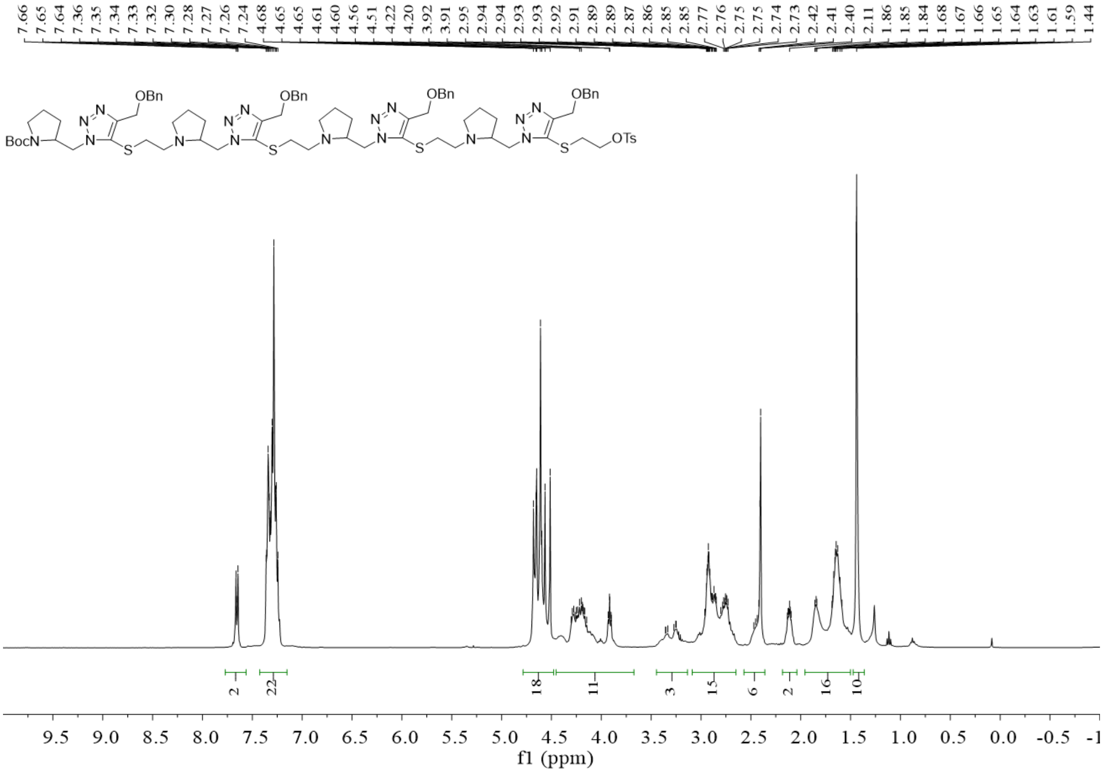

Figure S44. ^{1}H NMR spectra of **6b**-OTs.

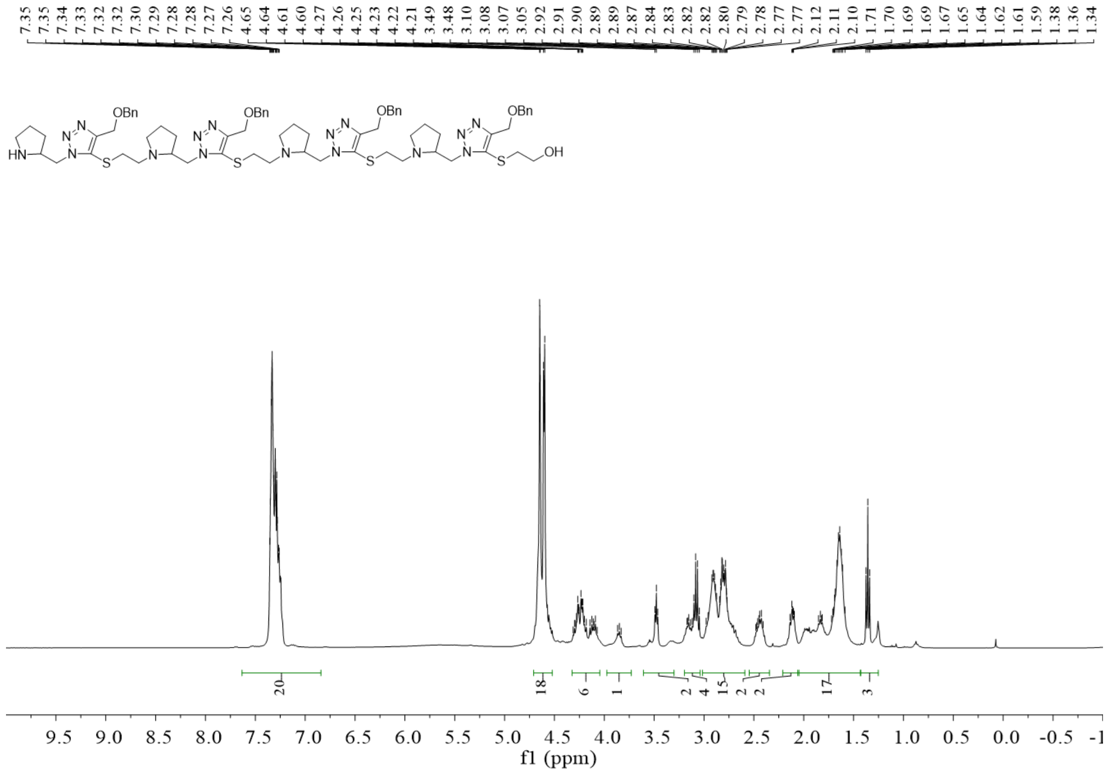

Figure S45. ^{1}H NMR spectra of **6b**-H.

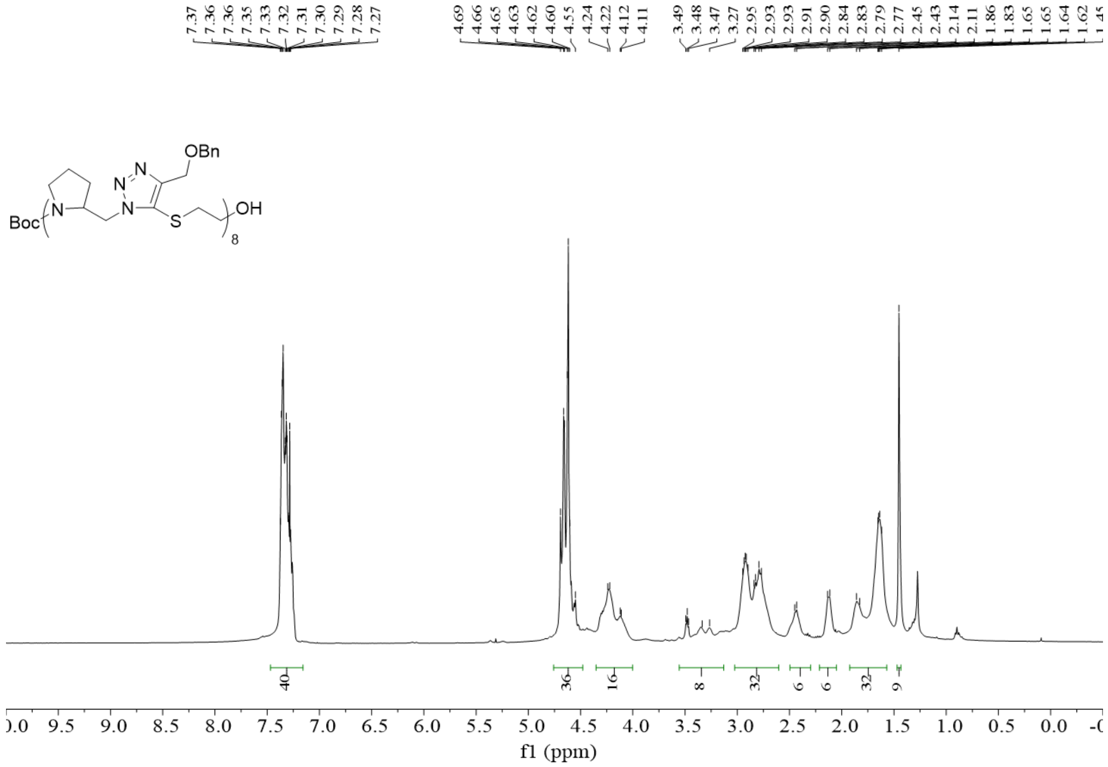

Figure S46. ^{1}H NMR spectra of **8b**

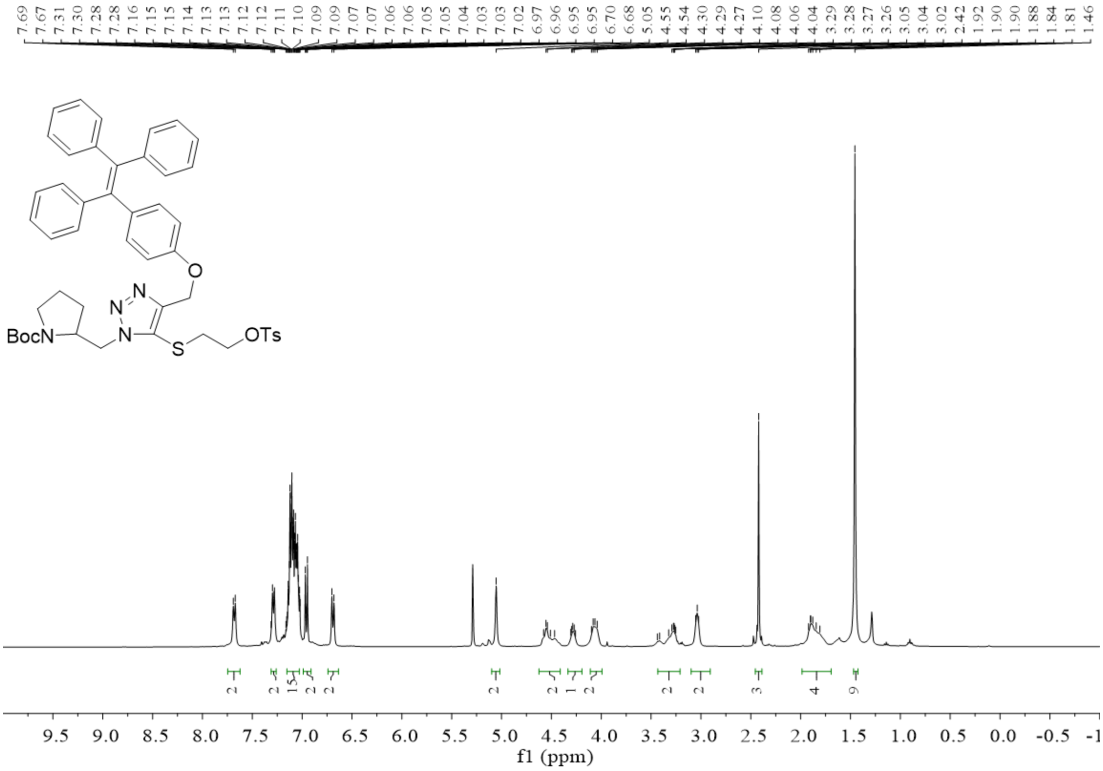

Figure S47. ^{1}H NMR spectra of **4c**-OTs.

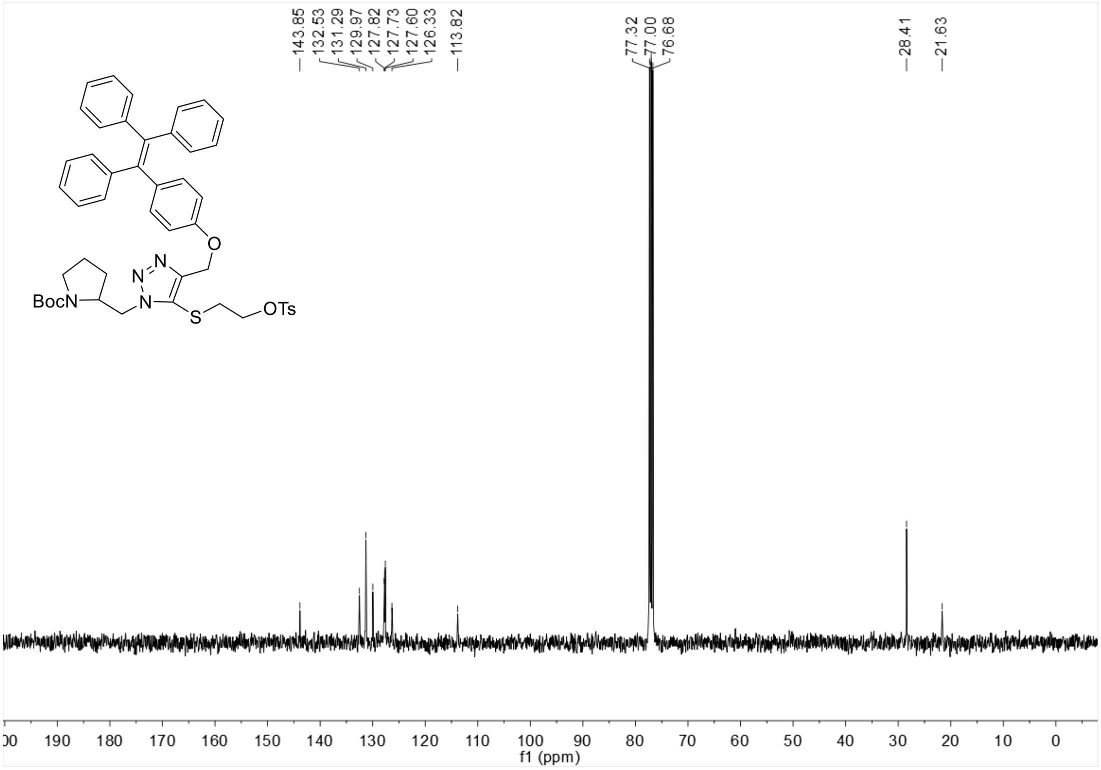

Figure S48. ^{13}C NMR spectra of **4c**-OTs.

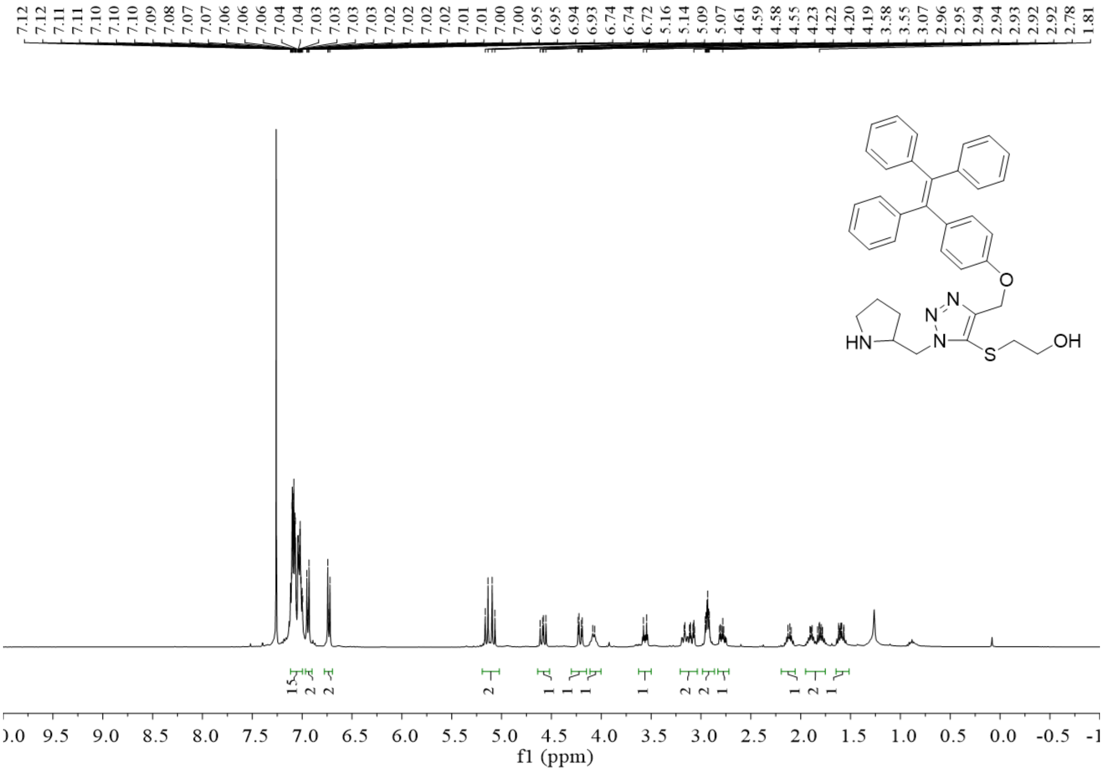

Figure S49 ^{1}H NMR spectra of **4c**-H.

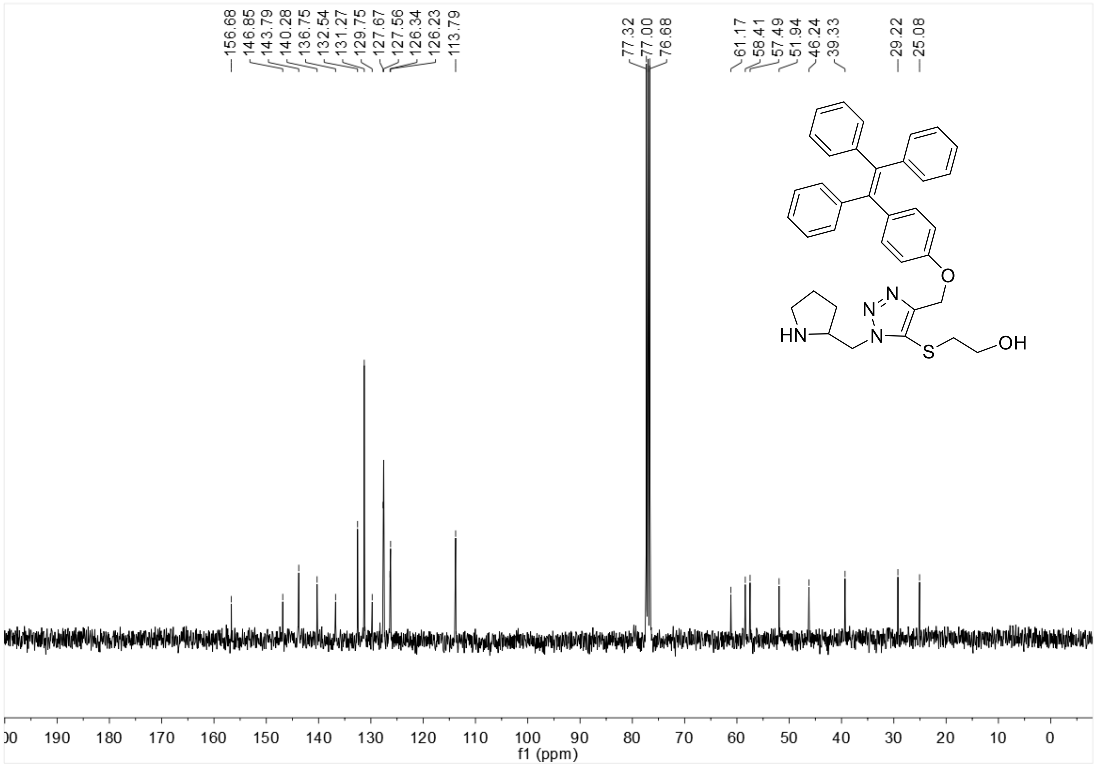

Figure S50. ^{13}C NMR spectra of **4c**-H.

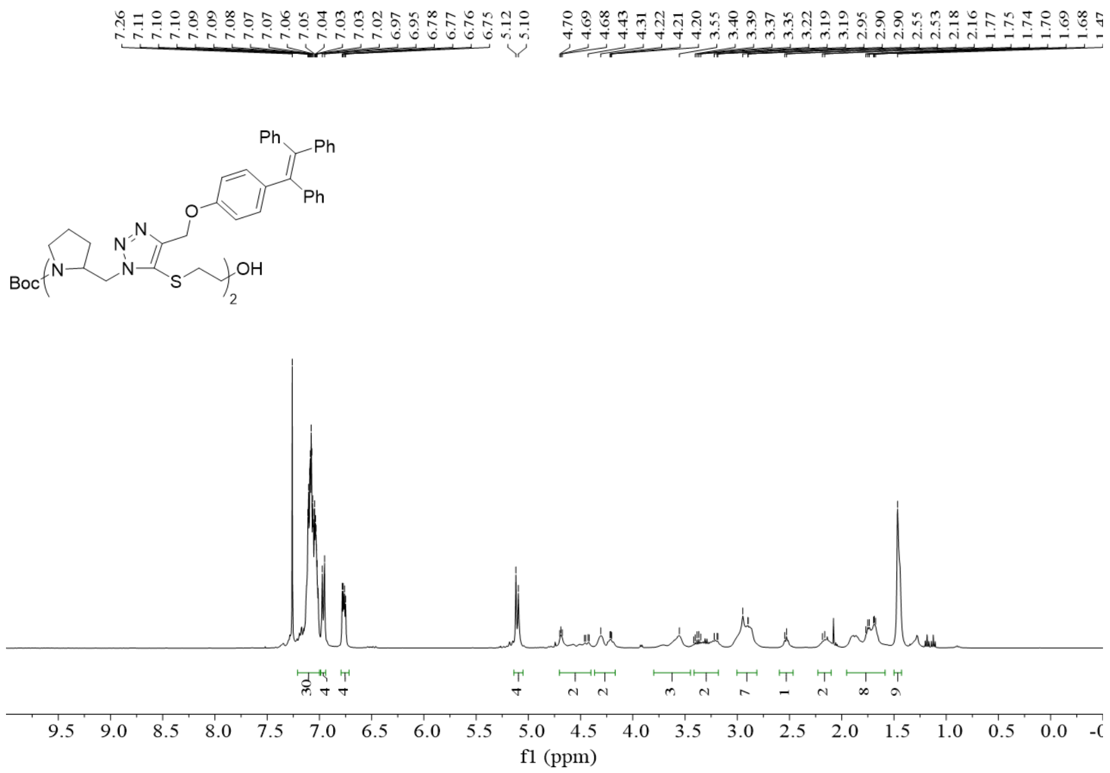

Figure S51. ^{1}H NMR spectra of **5c**.

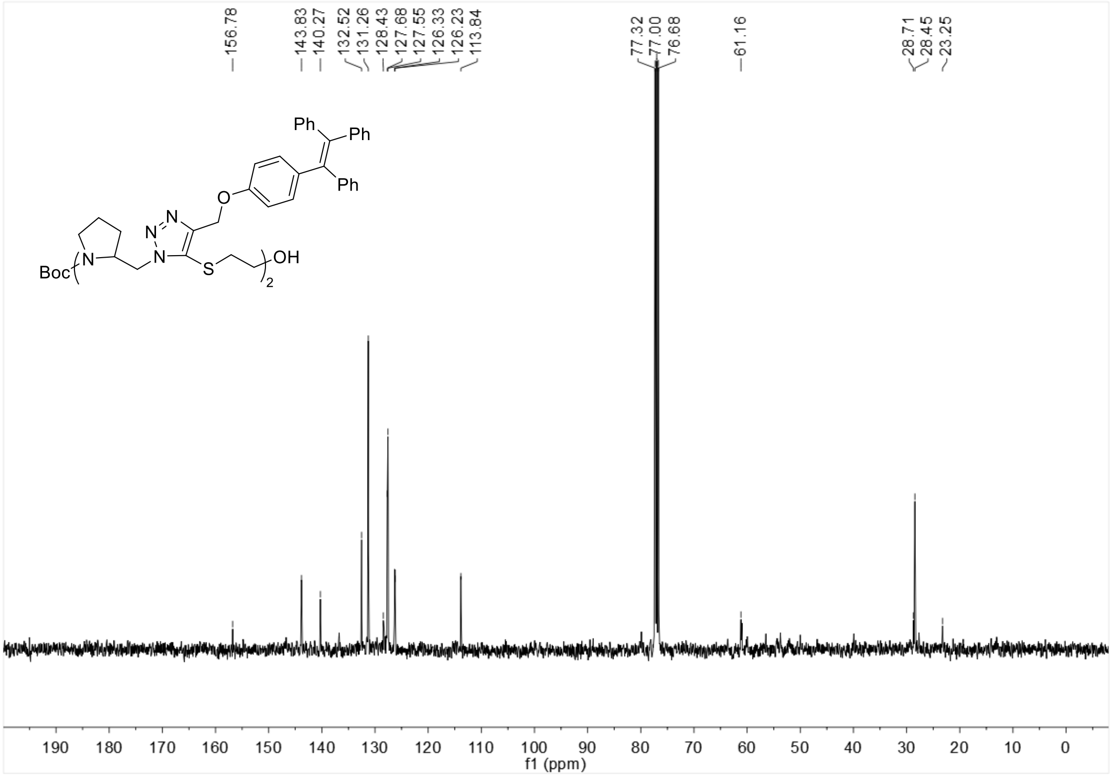

Figure S52. ^{13}C NMR spectra of **5c**.

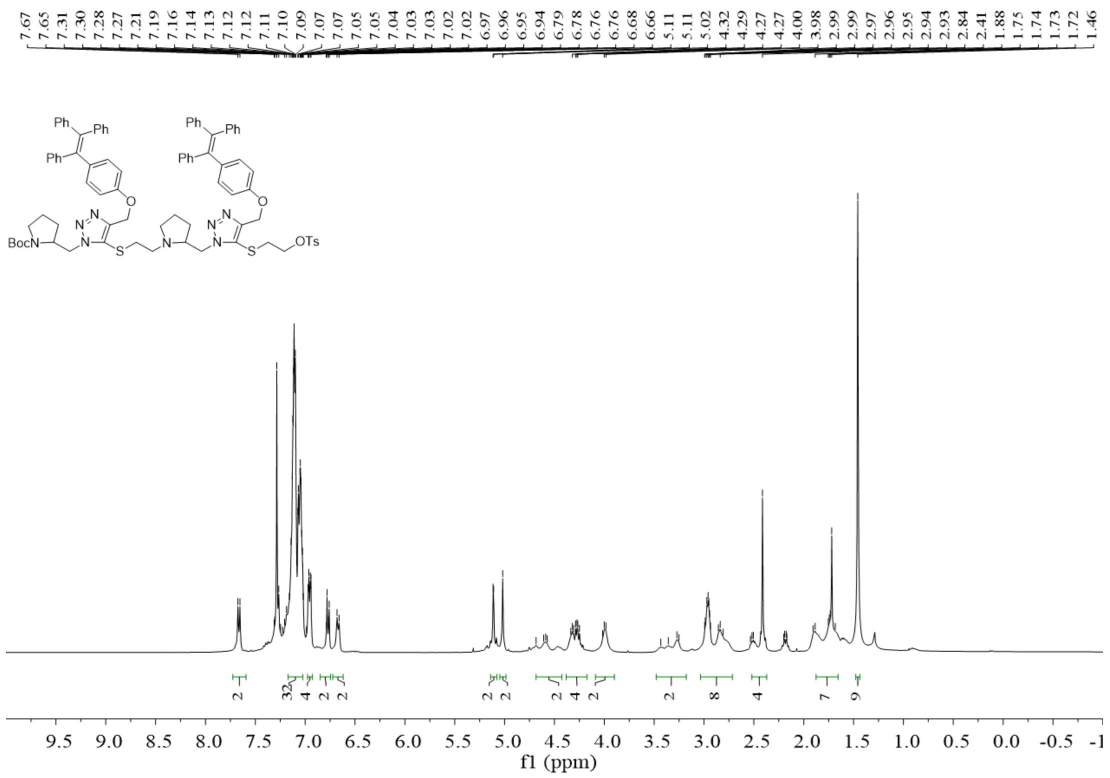

Figure S53. ^{1}H NMR spectra of **5c**-OTs.

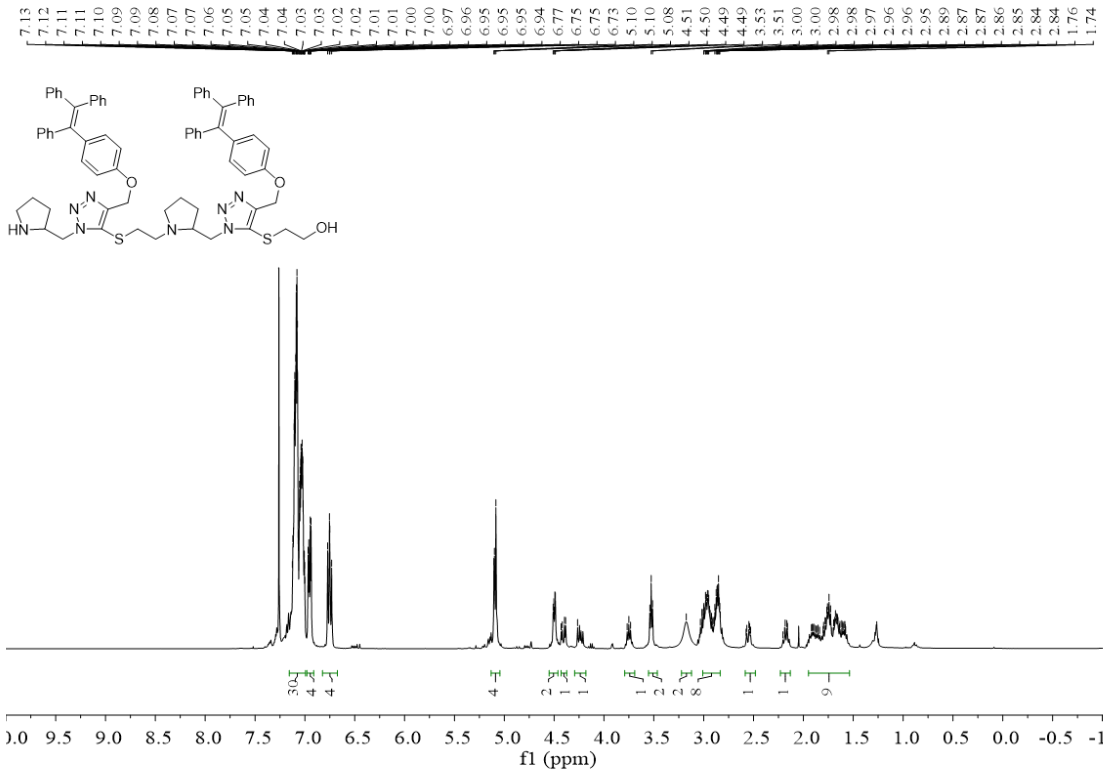

Figure S54. ^{1}H NMR spectra of **5c**-H.

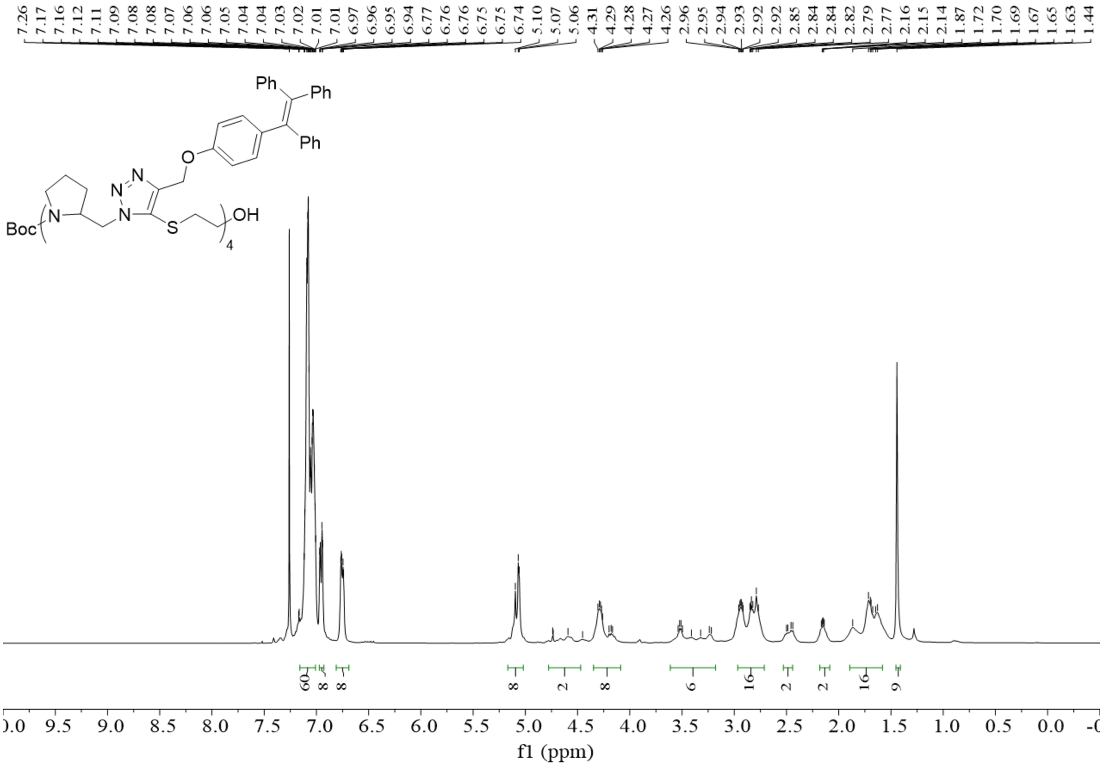

Figure S55. ^{1}H NMR spectra of **6c**.

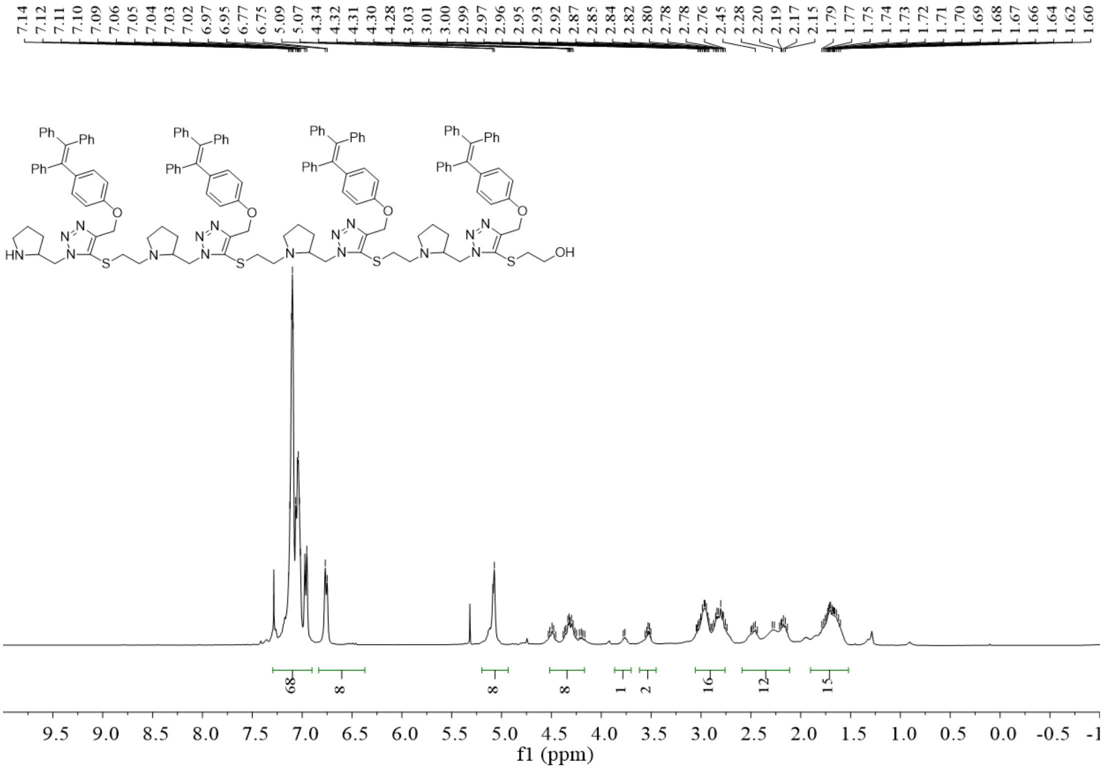

Figure S56. ^{1}H NMR spectra of **6c**-H.

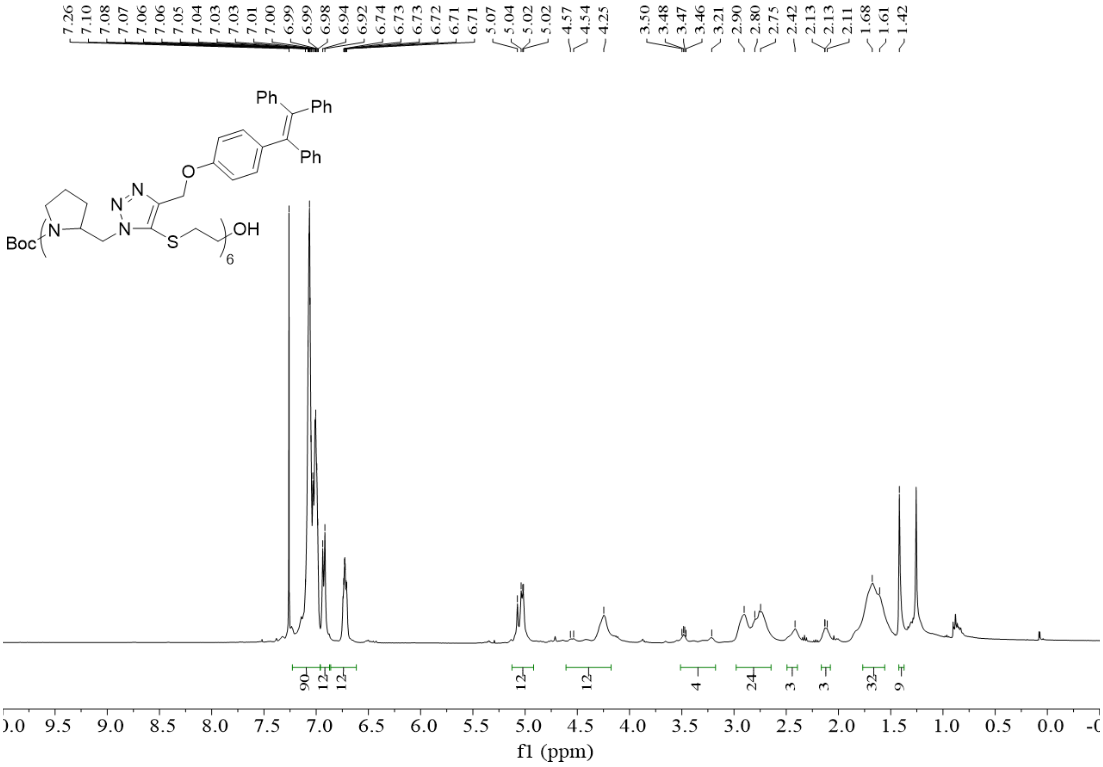

Figure S57. ^{1}H NMR spectra of **7c**.

II. MS spectra

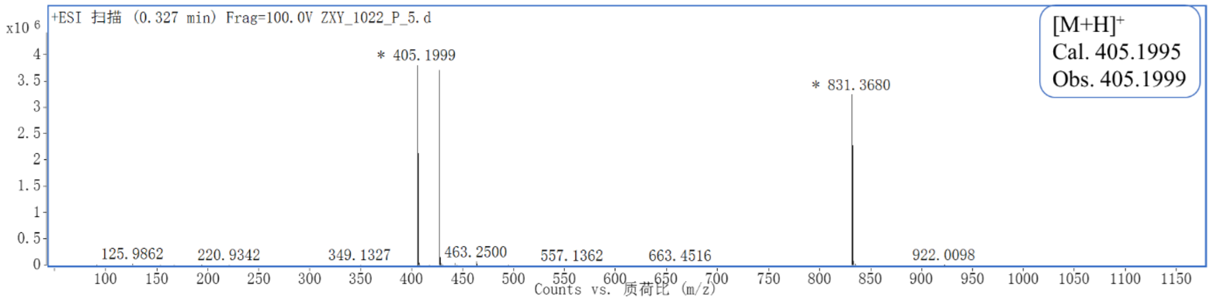

Figure S58. HRMS spectra of **4a**.

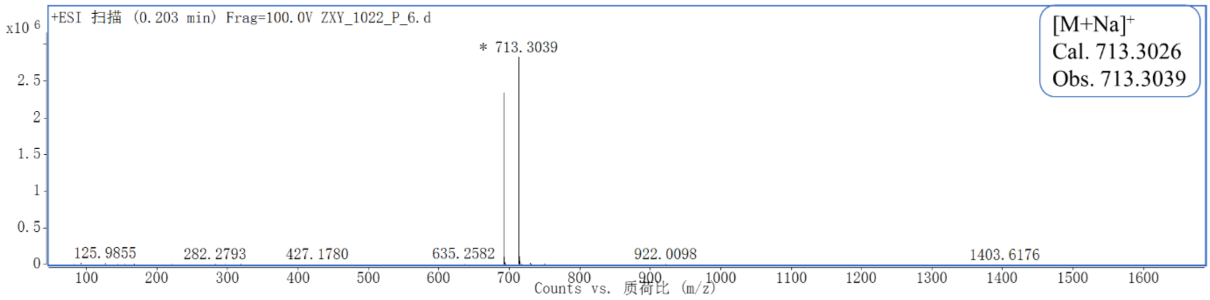

Figure S59. HRMS spectra of **5a**.

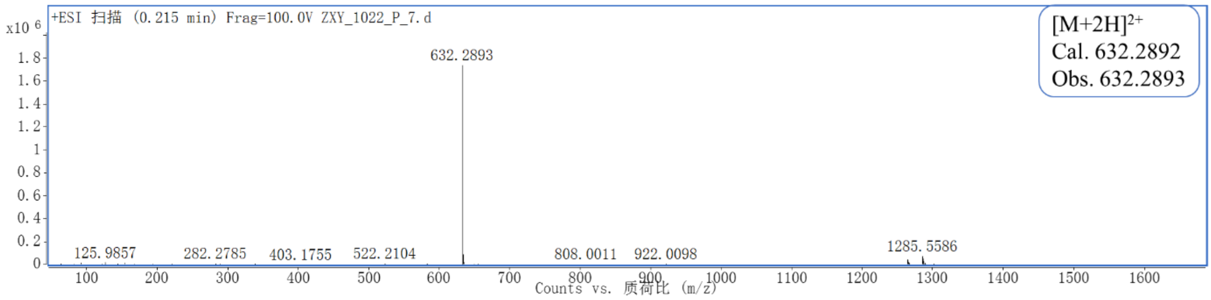

Figure S60. HRMS spectra of **6a**.

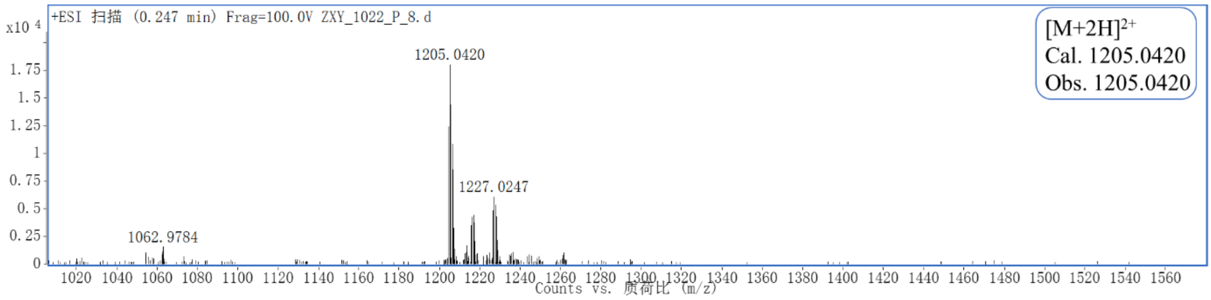

Figure S61. HRMS spectra of **8a**.

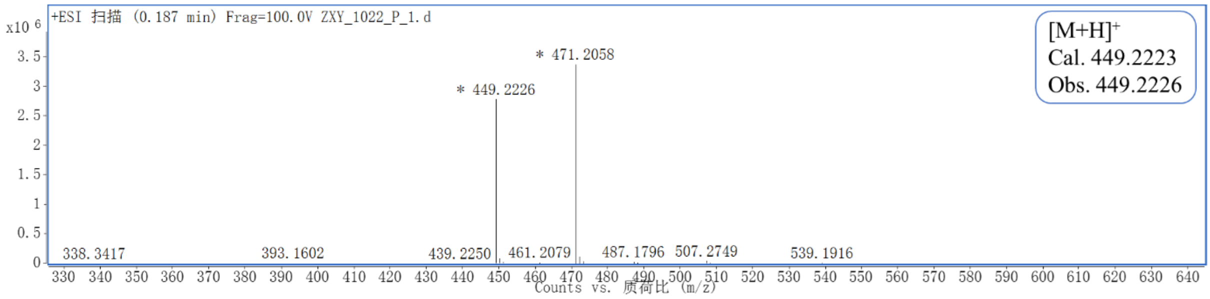

Figure S62. HRMS spectra of **4b**.

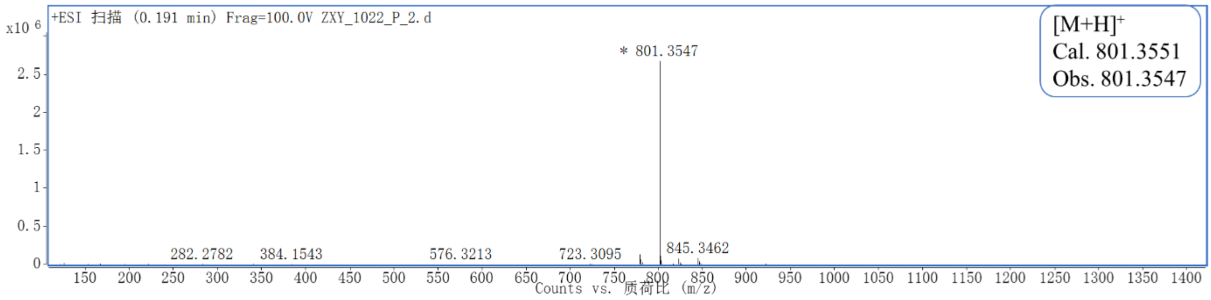

Figure S63. HRMS spectra of **5b**.

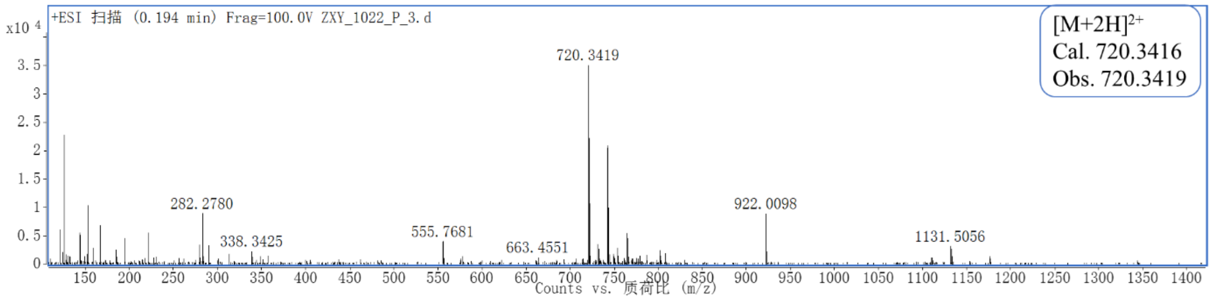

Figure S64.HRMS spectra of **6b**.

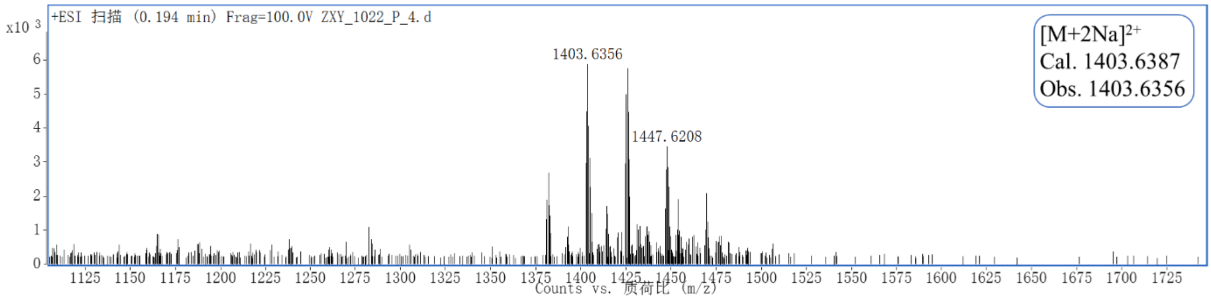

Figure S65. HRMS spectra of **8b**.

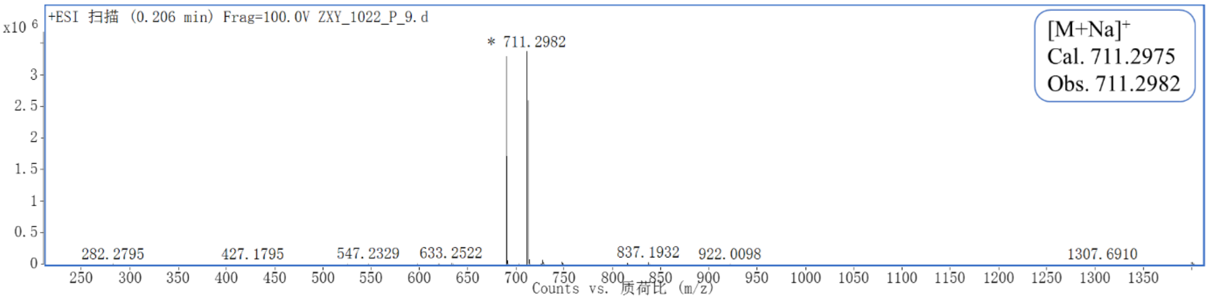

Figure S66. HRMS spectra of **4c**.

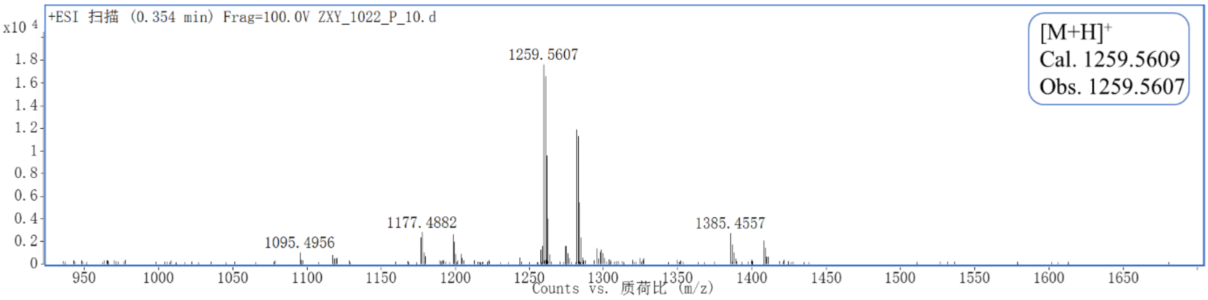

Figure S67. HRMS spectra of **5c**.

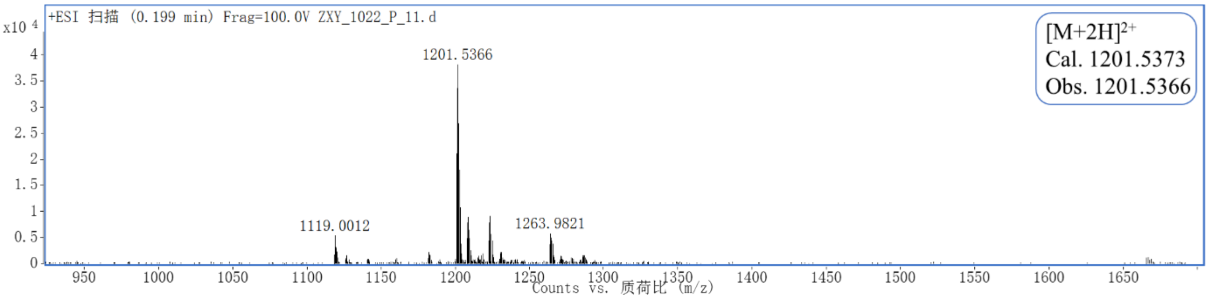

Figure S68. HRMS spectra of **6c**.
